# Heart rate supports accelerometry as a reliable predictor of energy expenditure in bats

**DOI:** 10.1242/jeb.251521

**Published:** 2026-10-22

**Authors:** Travis D. Bayer, M. Graciela Aguilar, Leslye M. Barría, Luisa F. Gómez-Feuillet, James P. Lee, M. Teague O'Mara

**Affiliations:** ^1^ Pathos Wildlife, Fiskeville, RI 02823, USA; ^2^ Department of Natural Resources Science, University of Rhode Island, Kingston, RI 02881, USA; ^3^ Department of Biological Sciences, Southeastern Louisiana University, Hammond, LA 70402, USA; ^4^ Veterinary Clinical Science Department, Louisiana State University, Baton Rouge, LA 70803, USA; ^5^ Smithsonian Tropical Research Institute, Ancon, 0843-03092, Panamá; ^6^ Universidad de Costa Rica, Sede Rodrigo Facio, San José, San Pedro 11501-2060, Costa Rica; ^7^ Bat Conservation International, Austin, TX 78746, USA; ^8^ Department of Migration, Max Planck Institute of Animal Behavior, Radolfzell, 78315, Germany

**Keywords:** Bio-logging, Vectorial dynamic body acceleration, *Phyllostomus hastatus*, Bat energetics, Flight energetics, Movement ecology

## Abstract

Measuring the energetic cost of behavior is essential for understanding decision-making in wild animals. This is particularly true for bats, where life near their energetic ceiling has resulted in a diversity of behaviors to compensate for social and environmental unpredictability. Although GPS and tri-axial accelerometry have improved movement-based estimates of energy expenditure in the field, complex flight kinematics in bats may violate key assumptions accelerometry-based estimates rely on. To test whether energy expenditure is predicted by acceleration-based movement in bats, we deployed heart rate loggers alongside GPS with tri-axial accelerometers on free-ranging greater spear-nosed bats (*Phyllostomus hastatus*) in Panamá. We hypothesized that heart rate would positively reflect increases in vectorial dynamic body acceleration (VeDBA), a commonly used proxy for energy expenditure, and that including information on behavior would enhance this relationship. We also hypothesized that heart rate and VeDBA would reflect theoretical speed–power curves of flight. We found that VeDBA had a strong positive relationship with heart rate, and model accuracy improved when behavioral states of flying and not flying were defined. Additionally, incorporating behavioral duration and sequence number improved predictions, showing that temporal context and activity patterns further refine estimates of energy expenditure. The relationship between VeDBA and airspeed resembled the mechanical power curve previously estimated for *P. hastatus*, supporting the relevance of aerodynamic theory in interpreting bat energetics. Overall, acceleration-based estimates reliably predict energy expenditure in free-ranging *P. hastatus*, and incorporating behavior improves model accuracy and ecological relevance, providing a powerful tool for understanding energy allocation in wild bats.

## Teaser

**Highlighted Article:** Simultaneous measurements with heart rate demonstrate that tri-axial accelerometry reliably predicts energy expenditure in free-ranging bats, revealing that individuals adjust their airspeed during long commutes to minimize energetic costs.

## INTRODUCTION

Energy serves as the currency of life and influences nearly every aspect of biology (Brown et al., 2004; Butler et al., 2004; Tomlinson et al., 2014), and successfully balancing energy intake and expenditure is crucial to success (Brown et al., 2004; Jeanniard-du-Dot et al., 2017a; Stephens and Krebs, 1986). As a result, this creates a metabolic constraint that can influence behaviors aimed at maximizing food intake while minimizing energy costs (Brown et al., 2004; Butler et al., 2004; Stephens and Krebs, 1986; Tolkamp et al., 2002). This challenge is particularly evident in bats, which live near their metabolic ceiling, rely on an energetically costly form of locomotion, and depend on ephemeral food resources. Many species demonstrate remarkable flexibility in their energy distribution by adjusting heart rates (O'Mara et al., 2017), movement decisions (Calderón-Capote et al., 2024a; O'Mara et al., 2021) and behavioral strategies (Egert-Berg et al., 2018; Wilkinson and Boughman, 1998) to satisfy their energy requirements in a constantly changing environment. Understanding how bats navigate metabolic challenges and the role of energy distribution in their ecology and evolution is fundamental to explaining the relationship between ecosystem dynamics and the adaptations of species to their environments (Nathan et al., 2008; O'Mara et al., 2019; Pretzlaff et al., 2010; Rubalcaba et al., 2022; Shepard et al., 2013). This requires effective methods to measure or estimate energy distribution across different behaviors and time scales (Shepard et al., 2013; Wilson et al., 2012). However, quantifying energy expenditure in free-living animals can be challenging. Numerous methods for measuring or estimating energy expenditure have been developed (Green, 2011; Wilson et al., 2006; Butler et al., 2004; Hicks et al., 2017; Sutton et al., 2023), but much of this work has relied on captive use in a laboratory setting or invasive procedures, requires substantial resources, or provides estimates at very broad temporal scales.

One less-invasive method that is field deployable uses tri-axial accelerometers to measure an animal's dynamic body acceleration (Gleiss et al., 2011; Qasem et al., 2012; Wilson et al., 2006, 2020). In addition to estimating energy expenditure, accelerometry can also remotely identify animal behaviors, allowing researchers to link fine-scale activity patterns with energetic costs in the field (Brown et al., 2013; Hicks et al., 2017; Patterson et al., 2019; Shepard et al., 2008; Williams et al., 2014). By summing data from all three axes, vectorial dynamic body acceleration (VeDBA) or overall dynamic body acceleration (ODBA) has been shown to be an effective estimate of short-term energy expenditure across various taxa (Allen et al., 2022; Brownscombe et al., 2018; Elliott et al., 2013; Enstipp et al., 2011; Gleiss et al., 2011; Halsey et al., 2009; Hetem et al., 2025; Jeanniard-du-Dot et al., 2017b; Miwa et al., 2015; Pagano and Williams, 2019; Qasem et al., 2012; Rezende et al., 2023 preprint; Robson et al., 2016; Stothart et al., 2016; Sutton et al., 2023; Udyawer et al., 2017; Williams et al., 2014; Wilson et al., 2020). However, muscle efficiency varies with different modes of locomotion and the medium being traversed (Gleiss et al., 2011; Laich et al., 2011; Wilson et al., 2020). Understanding movement ecology requires consideration of the physiological constraints that govern how animals allocate energy (Hetem et al., 2025). As a result, it is necessary to give thought to factors such as the method of locomotion and how it interacts with the environment when determining whether a proxy accurately reflects energy expenditure (Gleiss et al., 2011; Hicks et al., 2017). This raises uncertainty about the reliability of estimates such as VeDBA in animals with complex locomotion kinematics or those moving through viscous or resistant mediums, as it is heavily based on terrestrial estimates of acceleration-based energy expenditure. Without further verification, there is a substantial risk of errors in estimating energy expenditure, potentially leading to inaccurate conclusions about chiropteran behavior, ecology, physiology and conservation strategies.

In this study, we aimed to describe how accelerometry-based metrics of energy expenditure reflect measures of heart rate throughout a foraging night in the greater spear-nosed bat (*Phyllostomus hastatus*). The greater spear-nosed bat is an omnivorous neotropical bat species that lives at the limit of its metabolic capacity, surviving on the energy provided by the day's feeding returns (O'Mara and Dechmann, 2023). These metabolic requirements are intensified in populations roosting in the caves on Isla Colón, Panamá, where individuals frequently fly more than 25 km to foraging sites every night (Calderón-Capote et al., 2024a; O'Mara and Dechmann, 2023). To fulfill these energetic demands, *P. hastatus* alters their diet to match the seasonal changes and resulting resource shifts, switching between fruit (e.g. cecropia fruit) in the wet season and the flower nectar (e.g. balsa flowers) in the dry season, as well as supplemental insects and other small vertebrates (Wilkinson and Boughman, 1998). This dependence on ephemeral resources can result in a larger diversity of foraging strategies (Egert-Berg et al., 2018; Kohles et al., 2024; O'Mara and Dechmann, 2023; Wilkinson, and Boughman, 1998, 1999) and, in turn, dynamic energy costs across a night or season (Calderón-Capote et al., 2024a; Kohles et al., 2024; O'Mara and Dechmann, 2023). Changes in these movement-based decisions may result from the constantly shifting cost versus return of each foraging night, and by estimating energy expenditure, we can provide context to their movement choices and associated costs (Kohles et al., 2024; O'Mara et al., 2017).

By outfitting 12 greater spear-nosed bats on Isla Colón, Panamá, with heart rate loggers and tri-axial accelerometers, we were able to measure heart rate and acceleration simultaneously, which allowed us to directly compare these proxies of energy expenditure in free-ranging bats for the first time. To evaluate the effectiveness of tri-axial accelerometers in estimating energy expenditure, we used heart rate data to assess how well VeDBA reflected the energy use of individual bats. Direct measurements of heart rate provided detailed information into energy expenditure with high temporal resolution (Bishop and Spivey, 2013; Brown et al., 2022; Butler et al., 2004; Green, 2011; Green et al., 2009). Using generalized additive mixed models (GAMMs), we constructed models that predicted heart rate based on accelerometry-based metrics of energy expenditure. Given that the cost of movement can make up a substantial proportion of an animal's daily energy budget (Jeanniard-du-Dot et al., 2017c; Karasov, 1981; Pontzer, 2025), we hypothesized that VeDBA would positively reflect increases in heart rate. We also hypothesized that incorporating behavioral information would improve the accuracy of our energy estimates, as higher activity levels are associated with increases in energy expenditure. Finally, because airspeed is an important predictor of metabolic flight requirements (O'Mara and Dechmann, 2023; Rayner, 1999; Pennycuick, 2008), we hypothesized that airspeed would reflect changes in heart rate consistent with previously estimated mechanical power curves for this species. As a result, we expected that during long-distance commutes, bats would fly within an energetically optimal range of flight speeds.

Quantifying how an animal's energy expenditure varies across time and space can provide critical insights for conservation by allowing us to measure the associated energy costs imposed by changing landscapes and resources (Ellis-Soto et al., 2025; Madliger et al., 2021; Shepard et al., 2013; Wilson et al., 2012). Ultimately, developing a reliable, less-invasive method to estimate energy expenditure in free-living bats will help us explore how individual bats make movement decisions and better understand how these animals respond to ongoing environmental challenges.

## MATERIALS AND METHODS

### Study overview and design

All data used in this study were collected from 12 greater spear-nosed bats [*Phyllostomus hastatus* (Pallas 1767)] from a single roosting group, consisting of 11 females and 1 harem male (Table S1) (Calderón-Capote et al., 2024a,b). All bats were determined to be non-reproductive. The roosting group was captured in a cave (‘La Gruta’) on Isla Colón, Bocas del Toro, Panamá (Fig. 1), using an elongated pole and hoop net. Based on previous work of this species in Trinidad (Wilkinson and Boughman, 1998), roosting groups consisted of individuals co-roosting in small depressions in the cave's ceiling. All bats were caught as a group in one capture event, and individuals were placed in breathable cotton drawstring bags and tied shut until being removed for processing. We recorded the mass of each bat to the nearest gram and measured their forearm length to the nearest 0.01 mm. Each bat was implanted with a passive integrated transponder (PIT) tag subcutaneously (Trovan ID −100, Euro ID, Weilerswist, Germany).

**Fig. 1.**
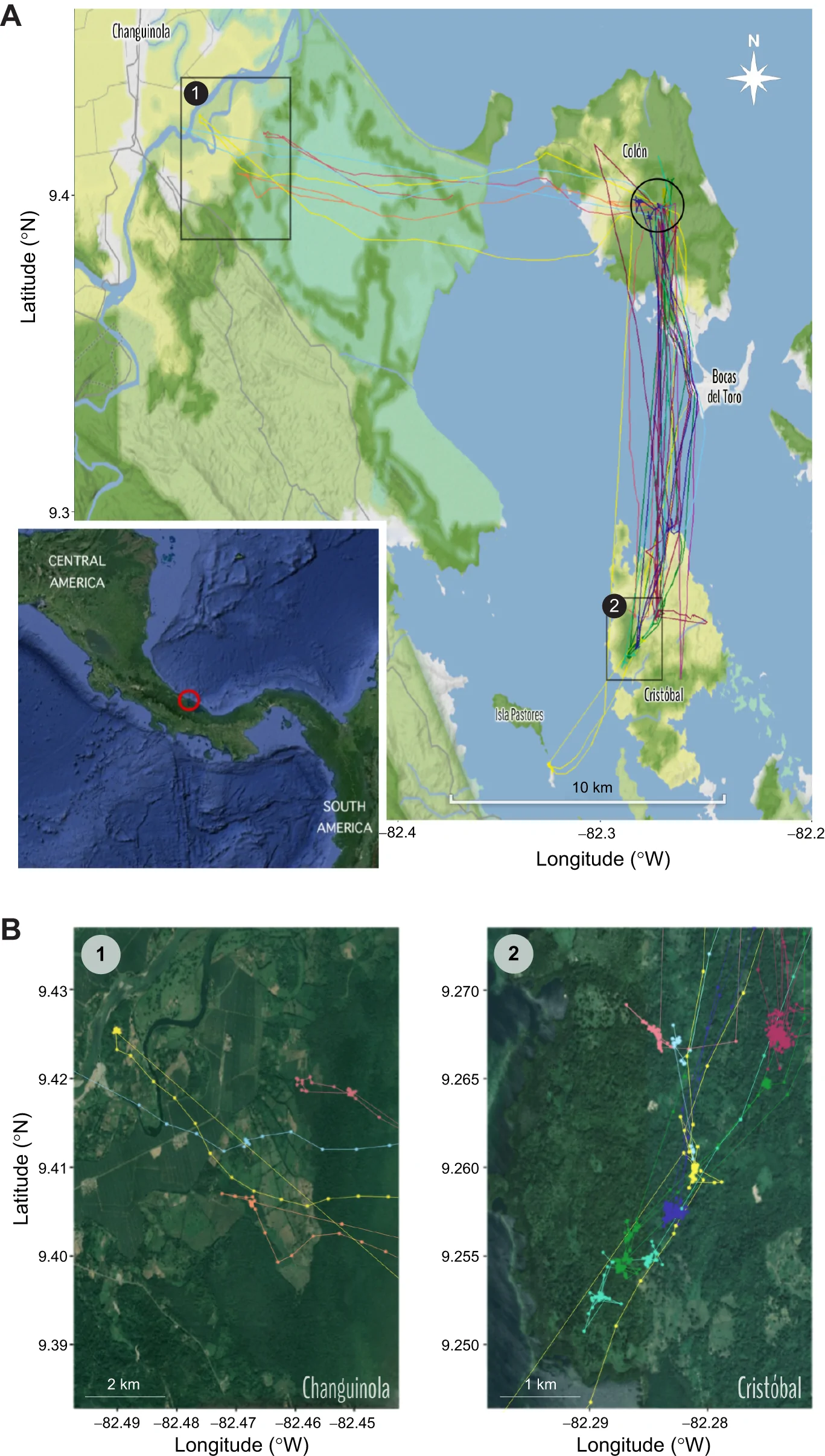
**Tracking summary across six nights showing foraging behaviors of *Phyllostomus hastatus*.** (A) Tracked routes. Different colors indicate individual bats. The black circle indicates the location of La Gruta cave, where the bats roosted. Numbered black boxes indicate main foraging aggregations. Outset shows the isthmus connecting North and South America, with Isla Colón marked by a red circle. (B) Numbered insets enlarge the primary foraging aggregations, detailing each foraging cluster's location.

### Ethical statement

This study was approved by the Ministerio del Ambiente, Panamá (SE/A-38-2020) as well as the Animal Care and Use Committee at the Smithsonian Tropical Research (SI-23016-1) and conformed to the ASAB/ABS Guidelines for the Use of Animals in Research.

### Bio-logger attachments and programming

#### Heart rate and temperature logger

An integrated physiology logger (DST micro-HRT, 3 g, Star:Oddi, Gardabaer, Iceland) was used to measure heart rate and body temperature from individual bats. Each logger was programmed to record body temperature and a 1 s measurement of heart rate at 600 Hz every 60 s, with a full ECG recorded once per hour. Each heart rate recording received a quality score (0–3, best–worst) based on the manufacturer's scoring algorithm. Loggers were implanted subcutaneously on the dorsum of each bat, laterally of the midline, under sterile conditions. Anesthesia was induced with 5% isoflurane delivered via a facemask (SomnoSuite, Kent Scientific, Litchfield, CT, USA) and maintained at 2% isoflurane throughout the procedure. A heating pad was used to reduce the risk of hypothermia. At the end of each procedure, a topical spray of neomicin and clostebol [Unipharm (International) AG, Chur, Switzerland] was applied to the implantation site to promote rapid healing. After a physical examination, each bat received a 0.2 mg kg^−1^ dose of meloxicam (OstiLox) and 8 ml kg^−1^ of NaCl fluids subcutaneously. On average, the entire procedure took approximately 15 min. The animals were then maintained in recovery bags until fully awake, and all members of the harem were released together at their roost. To remove the loggers, all bats underwent the same anesthesia procedure and examination. All animals exhibited a clean and healing surgical site at recapture. Recaptured bats received a 0.15 mg kg^−1^ dose of meloxicam (OstiLox), 10 ml kg^−1^ of NaCl fluids subcutaneously and a 5 mg kg^−1^ dose of enrofloxacin (Enroflox 10). The animals were again maintained in recovery bags until fully awake and finally released at their roost.

#### GPS and tri-axial accelerometer

After recovery from the procedure, all bats were outfitted with an epoxy-coated GPS and tri-axial accelerometer data logger (Axy-Trek Mini, TechnoSmart, Rome, Italy) that was attached to the dorsum, caudally to the scapulae, and cranially to the heart rate logger. The combined mass of the GPS and heart rate loggers was 7.8 g, which was 6.56±0.5% of mean±s.e.m. body mass (range: 5.3–7.1%), within the typical range of loggers carried by bats (O'Mara et al., 2014; Meierhofer et al., 2025). The GPS tags were programmed to gather location data every 1 min, between the hours of 18:00 to 05:00 h (Eastern Standard Time). While actively tracking, a satellite search window of 2 min was set to obtain a GPS fix. During instances of poor reception, the GPS tags entered a low-energy sleep mode for 15 min before resuming a satellite search. Performance of each GPS tag varied due to the location of their roost (within a cave), their foraging sites being located in presumably dense forest, and resting under thick canopies. Tri-axial accelerometers were programmed to sample continuously at a frequency of 25 Hz at 10 bits with a dynamic range of ±8 ***g***. Tags were positioned so the *x*-axis correlated to sway (left–right), the *y*-axis to surge (forward–backward) and the *z*-axis to heave (up–down). Bats were recaptured using the same methods on the sixth day of deployment shortly after 06:00 h and all loggers were recovered. To avoid unusual behavior caused by stress from the newly attached devices, the first night of data was excluded from our data analyses. We also removed one bat from our analyses (GPS ID: Phyl 30) as a result of inactivity, providing us with data from 10 females and 1 male, with each bat tracked for 4.10±1.16 nights (range: 1.8–5 nights), for a total of 496.32 total tracking hours after these adjustments. Accelerometers and heart rate loggers sampled continuously for the duration of the study and recorded 1216.12 h for each logging device (Bayer et al., 2026).

Wind data were recorded at 15-min intervals at an automated weather station (9°21′04″N, 82°15′29″W) managed by the Physical Monitoring Program at the Smithsonian Tropical Research Institute's Bocas del Toro field station. Wind speed and direction measurements were taken every 10 s using an RM Young Wind Monitor Model 05103. At the end of each 15-min interval, the mean wind speed and direction were calculated. Following O'Mara and Dechmann (2023), we calculated ground speed (movement relative to the ground) and bearing for successive time points using the move package (https://CRAN.R-project.org/package=move). To calculate airspeed (movement relative to the moving air column), we annotated wind speed and direction for each GPS location using a weighted interpolation of the *U* and *V* components of the available 15-min wind data to match the higher temporal resolution of the GPS measurements (O'Mara et al., 2019, 2021; Safi et al., 2013). Wind support was calculated as the component of the wind vector in the direction of the bat's flight, where positive values represent tailwinds and negative values represent headwinds, and is reported in m s^−1^. Crosswind was calculated as the magnitude of the wind vector perpendicular to the travel direction. Airspeed was then calculated as the square root of (ground speed–wind support)^2^+crosswind^2^.

### Data analysis

#### Acceleration

All data were processed and analyzed in R version 4.2.2 (https://www.r-project.org/), and summaries are presented as means±s.d.

We calculated static acceleration, dynamic acceleration, vectorial dynamic body acceleration (VeDBA) and pitch from the raw tri-axial acceleration. Static acceleration (*S_x_*, *S_y_*, *S_z_*) was calculated for each axis as:

(1) $$S=\frac{\Sigma_{x}}{n},\;\;\frac{\Sigma_{y}}{n},\;\;\frac{\Sigma_{z}}{n},$$

where *n* is the sampling frequency. Using the static acceleration, pitch (θ) was calculated as:

(2) $$\theta =\mathrm{ta}n^{-1}\left(\frac{S_{x}}{\sqrt{S_{y}^{2}+S_{z}^{2}}}\right)\times \frac{180}{\pi }.$$

Dynamic acceleration (*D_x_*, *D_y_*, *D_z_*) was calculated for each axis as total acceleration minus static acceleration:

(3) $$D=x-S_{x},\;\;y-S_{y},\;\;z-S_{z},$$

where *x*, *y* and *z* are the acceleration measurements along the three axes, and the vectorial dynamic body acceleration (VeDBA) was calculated as:

(4) $$\mathrm{VeDBA}=\sqrt{D_{x}^{2}+D_{y}^{2}+D_{z}^{2}}.$$

Each axis of dynamic acceleration (*D_x_*, *D_y_*, *D_z_*) was summed, and we calculated a rolling average over a 2 s window to smooth the data for each acceleration metric. To bring the data to the same temporal resolution, estimates of VeDBA were summed to 1 min to match the heart rate sampling rate. See Table S3 for definitions of all symbols used in this study.

#### Heart rate

All raw heart rate data (Bayer et al., 2026) were first processed in Star:Oddi HRT Analyzer (Star:Oddi HRT Analyzer v.1.2.0, Mercury Application Software). Star:Oddi assigned quality index (QI) numbers ranging from 0 to 3 to each 1-min sample of data, where 0 represents the highest quality data and 3 the lowest. Following manufacturer's recommendations, to remove any sampling errors from the data frame, a data quality check was done by removing all samples with a QI of 2 and 3, as recommended by Star:Oddi (Star:Oddi HRT Analyzer v.1.2.0, User Manual, August 2023). We further used a randomly selected sample of 220 ECGs and compared manually calculated heart rate with automatically classified values and found a Pearson's correlation coefficient of 0.9995 on heart rates ranging from 110 to 600 beats min^−1^. Based on minimum and maximum heart rates for *P. hastatus* from previous literature (Thomas and Suthers, 1972), we removed heart rates above 900 beats min^−1^ and below 100 beats min^−1^. Together, these corrections resulted in a ∼22% loss of our data. We observed no bias towards high or low values in the heart rate QI. We estimated energy expenditure from heart rate data (Bishop and Spivey, 2013) by modeling oxygen consumption rate (*V*·_O_2__) as a function of heartbeat frequency (*f*_H_), heart mass (*M*_h_) and body mass (*M*_b_):

(5) $${\dot{V}}_{O_{2}}=0.0402\times M_{b}^{0.328}\times M_{h}^{0.913}\times f_{H}^{2.065}.$$

We estimated the heart mass of *P. hastatus* to be 1% of its total body mass (Canals et al., 2005; Thomas and Suthers, 1972). *V̇*_O_2__ was then converted to J min^−1^ (1 ml O_2_=21.11 J; *V̇*_O_2__×21.11) (O'Mara et al., 2017), and finally to kJ min^−1^.

#### Behavior classification

Acceleration data were manually inspected to section out durations of known behaviors using a basic understanding of flight physics and visual inspections of plotted data (Fig. S1). Distributions of metrics from identified behavior (not flying, flying) provided the minimum, maximum and most meaningful values for each variable during each behavior.

Metrics with clear and useful differences between each behavior were then selected for our decision tree (Patterson et al., 2019) (Fig. S2). A threshold towards the floor or ceiling of each behavior distribution, whichever was more likely, was chosen for the breakpoints in the tree. Depending on the quality of the threshold at identifying the unique behavior, flight metrics were hierarchically arranged in the tree. To smooth out potential errors in the behavioral classification model, behaviors occurring for less than 1 s were assigned to the previous behavior. Flight sequences were further categorized into commuting flights when applicable. Commuting flight was defined as any duration of flight lasting longer than 3 min and excluded the first and last 30 s of a flight sequence to account for landing and take-offs.

#### Modeling energy expenditure

Heart rate should reflect a high-fidelity estimate of energy expenditure (Bishop and Spivey, 2013; Thomas and Suthers, 1972). To determine possible relationships between heart rate and other estimates of energy expenditure, including VeDBA and airspeed, we constructed generalized additive mixed models (GAMMs). The relationship between energy expenditure and its predictors can vary across behaviors, environmental conditions and kinematic adjustments. Using GAMMs allows for more appropriate modeling of these complex, non-linear relationships and thus provides a more accurate representation of the factors influencing energy use.

Individual identity (tag ID) was included as a random intercept in all of our models. The structure of our baseline model was that heart rate was predicted by VeDBA (e.g. heart rate ∼VeDBA). This was used to determine any initial relationship between each metric and serve as a ‘type model’ to compare with our following models. Additionally, sustaining increased physical activity requires a continual supply of oxygen pumped to the working muscles, which would be reflected in an individual's elevated heart rate. So, we incorporated the effect of behavior (not flying, flying) into our models to understand how changes in heart rate reflect changes in VeDBA across two different levels of activity. We included the interaction between the behavior being performed and VeDBA using heart rate ∼(VeDBA): activity. Additionally, because the maximum rate of oxygen consumption works as an asymptote (Bishop and Spivey, 2013), we wanted to understand how this model would respond to an estimates of energy derived from our heart rate data (kJ min^−1^) as our dependent variable, instead of the heart rate data itself (Hicks et al., 2017). We structured this model as energy expenditure (kJ min^−1^) ∼VeDBA.

We used a multi-inference approach to select the best fitting models to describe the relationship between heart rate (and kJ min^−1^) and subsequent predictors. Likelihood ratio tests (LRTs) were used to assess whether the inclusion of specific smooth terms for predictor variables significantly improved model fit. The effective degrees of freedom (edf) were calculated for each predictor variable (smooth terms within the model). The edf reflects the complexity of the smooth terms in the model, and models with higher edf are more complex, with an edf of 1 reflecting a straight linear fit. The adjusted *R*^2^ (*R*^2^_adj_) of each model was calculated to measure how well the model explained the variability of the dependent variable. As more smooth terms were added and the complexity of our models increased, the *R*^2^_adj_ was penalized. Finally, we used Akaike’s information criterion (AIC) to evaluate the goodness-of-fit between models with the same dependent variables. AIC balances the trade-off between model complexity and goodness-of-fit. Our models with lower AIC values indicate a better fit, and we considered all models within two AIC units of the best fit models as equally likely.

## RESULTS

### Behavior and tracking summary

The bats (118.83±10.61 g) emerged from the roost at 20:50±1.16 h and returned at 05:23±1.86 h. During their commute, they encountered slight headwinds on departure and tailwinds on return, although wind conditions were generally low and variable throughout the foraging period. The average wind speed was 9.5±7.44 m s^−1^. Over the course of the study, each bat was tracked for 4.10±1.16 nights. This resulted in a total of 47.78 h of GPS data, 1512 h of accelerometry data and 1512 h of heart rate data. We identified 18 foraging and resting sites from eight individuals. On average, these bats traveled 17.73±4.14 km to reach off-island sites, including 5.96±2.05 km flown over the ocean. Bats primarily divided their time between two main aggregations of over-ocean foraging sites (Fig. 1). Although no bat was observed visiting the same patch of trees as another, either on the same or different nights, bats tended to distribute their foraging sites within relatively close proximity to one another, forming identifiable foraging aggregations, defined as GPS fixes less than 100 m apart. One aggregation was located on the mainland, approximately 2 km southeast from the city of Changuinola (site 1), while the other was on Isla Cristóbal (site 2), an island located directly south of Bocas del Toro. Bats were more likely to visit Isla Cristóbal (19 of 24 trips), which was 11–16 km away from their roost, than Changuinola (5 of 24 trips), which was 20–25 km away from the roost. One individual (HRL ID: 1863UHL; Tables S1, S2) was observed making additional stops at foraging sites on Isla Pastores, a small island 6.5 km southwest of the primary foraging sites on Isla Cristóbal. This bat alternated between these two sites on consecutive nights.

### Energy expenditure across time

The energy expenditure of *P. hastatus* was primarily distributed between local sunset and sunrise, shown by the shaded region in Fig. 2C. During the night (18:00–06:00 h), the average heart rate was 254.13±94.24 beats min^−1^ (range: 101–800 beats min^−1^) and the average VeDBA was 296.08±441.57 ***g*** min^−1^ (range: 23.61–3023.74 ***g*** min^−1^). In contrast, during the day (06:00–18:00 h), the average heart rate was lower, at 194.09±58.41 beats min^−1^ (range: 101–800 beats min^−1^), whereas the average VeDBA dropped to 105.44±100.03 ***g*** min^−1^ (range: 22.45–2064.59 ***g*** min^−1^). As airspeed increased, both heart rate and VeDBA gradually decreased, indicating an inverse relationship between energy expenditure and flight speed (Fig. 2B). Upon emerging from their roost, bats spent an average of 74.82±44.01 min commuting or foraging, and 297.40±145.97 min not flying. Activity peaked at approximately 21:00, 01:00 and 05:00 h, with average heart rate of 272.95±46.92 beats min^−1^ and VeDBA values of 403.62±146.65 ***g*** min^−1^ during these hours (Fig. 2C). Activity was lowest around 04:00 h, with a heart rate of 208.88±46.32 beats min^−1^ and VeDBA of 134.65±67.13 ***g*** min^−1^.

**Fig. 2.**
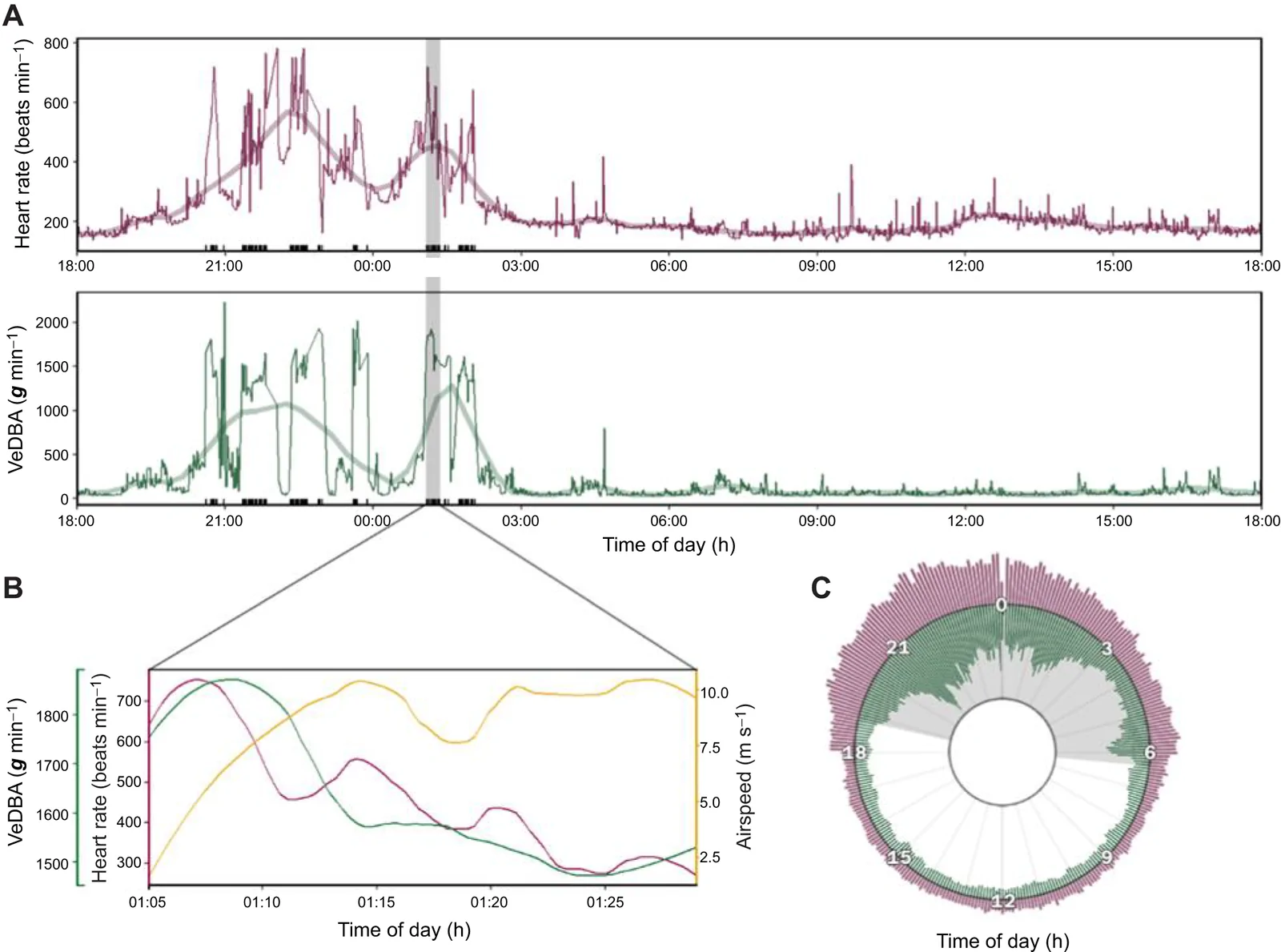
**Comparisons of energy metrics illustrating the temporal distribution of energy expenditure across different contexts.** (A) Heart rate (violet) and VeDBA (green) data across a 24-h period for GPS ID: Phyl 38. Black bars below each plot represent times of flight. (B) A 25 s segment of flight (indicated by the grey vertical band) compared the changes in heart rate and VeDBA with airspeed (gold). (C) A circular 24-h time plot showing the average distribution of heart rate and VeDBA across all bats by time of day, with shaded regions indicating the period between local sunset and sunrise.

### Energy expenditure across activity levels

Energy expenditure in *P. hastatus* was estimated through four metrics – heart rate (beats min^−1^), VeDBA (***g*** min^−1^) and airspeed (m s^−1^), as well as an additional metric of oxygen consumption (kJ min^−1^) – all of which met expectations across different activity levels and generally conformed with theory. VeDBA, heart rate and airspeed collectively reflected clear behavioral differences in energetic demands between non-flight behavior and flight in *P. hastatus* (Fig. 3; Fig. S3). VeDBA was lowest while not flying (mean: 130.66±135.18 ***g*** min^−1^), with brief spikes reflecting occasional activity (Fig. S3). In contrast, flight showed a broader and higher distribution (mean: 1426.52±366.46 ***g*** min^−1^), representing energetically intensive behaviors such as take-off and maneuvering, and marking a 991.4% increase from non-flight behavior. Heart rate followed a similar trend, increasing from a mean of 218.95±75.51 beats min^−1^ while not flying to 434.21±149.92 beats min^−1^ during flight (Fig. S3), leading to a 98.3% increase consistent with the greater cardiac demands of flight. Airspeed data revealed a bimodal distribution (Fig. 3), at higher (>6 m s^−1^; 57.76%) and lower (<3 m s^−1^; 31.53%) speeds, suggesting the use of distinct flight modes or strategies. The average flight speed (6.15±3.68 m s^−1^) was close to the species' estimated minimum power speed of 6.81 m s^−1^ (O'Mara and Dechmann, 2023). Nearly half of all flight data (48.68%) fell between this minimum power speed (6.81 m s^−1^) and the maximum range speed (11.0 m s^−1^), a range considered energetically optimal for *P. hastatus* (O'Mara and Dechmann, 2023).

**Fig. 3.**
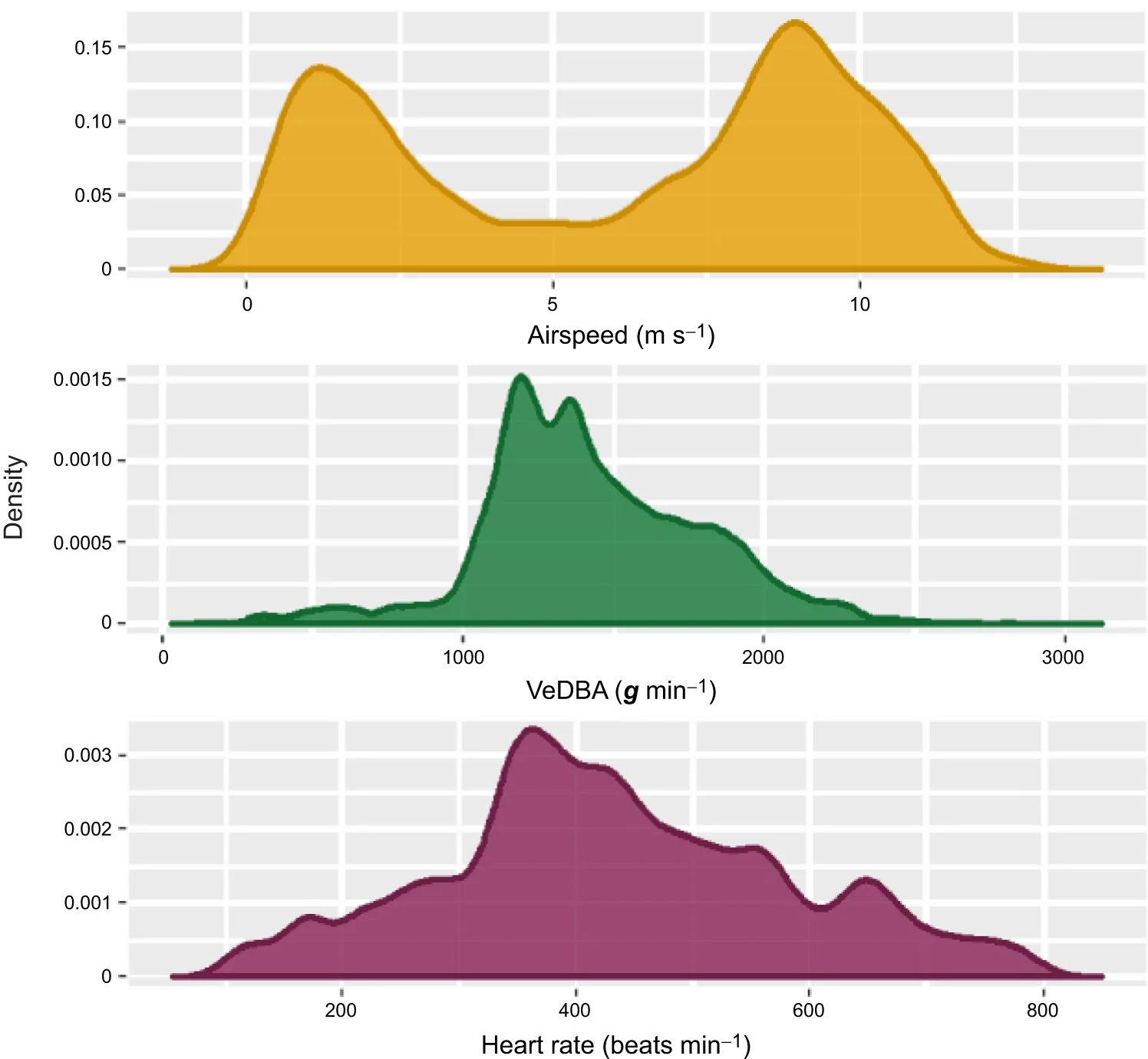
**Density plots illustrate the distribution of airspeed, heart rate and VeDBA data during periods of flight among bats.** Airspeed is indicated by gold, VeDBA by green and heart rate by violet.

### Modeling energy expenditure

Each model was constructed with different combinations of energy metrics and behavioral interactions to identify the most accurate representation of the relationship between heart rate and our estimates of energy expenditure. The results, including the model structures, statistics and relative performance values are presented in Table 1.

**Table 1.** GAMM results for the effect of vectorial dynamic body acceleration (VeDBA) on heart rate and energy expenditure in *Phyllostomus hastatus*

Model		*R* ^2^_adj_	edf	AIC
1	Heart rate ∼VeDBA	0.301	8.459	508,411
2	Heart rate ∼(VeDBA): flying (VeDBA): not flying	0.304	4.596 4.885	508,289
**5**	**Heart rate ∼(VeDBA): flying** **(VeDBA): not flying** **Duration** **Sequence number**	**0.441**	**4.398** **4.810** **8.960** **8.909**	**498,447**
3	Energy expenditure (kJ min^−1^) ∼VeDBA	0.286	4.529	−123,147
4	Energy expenditure (kJ min^−1^) ∼(VeDBA): flying (VeDBA): not flying	0.290	4.690 4.895	−123,071
6	Energy expenditure (kJ min^−1^) ∼(VeDBA): flying (VeDBA): not flying Duration Sequence number	0.368	4.587 4.850 8.935 8.796	−128,589
**7**	Heart rate ∼Airspeed	0.176	1.000	3188
9	Energy expenditure (kJ min^−1^) ∼Airspeed	0.150	1.000	−208
8	VeDBA ∼Airspeed	0.314	3.011	11,104

The model numbers shown correlate to the model numbers in the figures. Tag ID was included as a random effect for each model. Models are grouped by response variables to facilitate comparison among related analyses. The top performing model is in bold. Additional results shown for the effect of airspeed on heart rate, energy expenditure (kJ min^−1^) and VeDBA.

### VeDBA and heart rate relationship

To aid interpretation of model outputs, raw data colored by tag ID were plotted beneath each model. Displaying the observed values alongside the fitted smooth terms allows for a visual assessment of how well the models capture the underlying patterns in the data. Our first model (model 1, Fig. 4) examined the relationship between heart rate and VeDBA, with VeDBA included as a smooth term to allow for a non-linear effect. The model revealed a positive relationship between VeDBA and heart rate, where increases in dynamic body acceleration were associated with higher heart rate. The effective degrees of freedom (edf=8.459) indicates a non-linear association, and the model had an AIC of 508,411. A likelihood ratio test (LRT) comparing this model with a reduced model without the smooth term showed that including VeDBA as such significantly improved model fit (χ^2^_9.21_=75,475,521, *P*<0.001). Additionally, our sample included one larger male among predominantly female individuals (Table S1). Although individual-level variation was accounted for through the inclusion of tag ID as a random effect in all GAMMs, we refit model 1 excluding the male to ensure that he did not disproportionately influence model outcomes. Model fit was slightly reduced following removal (*R*^2^_adj_=0.291) compared with the full dataset (*R*^2^_adj_=0.301), indicating that inclusion of the male did not bias results and rather improved model outcome.

**Fig. 4.**
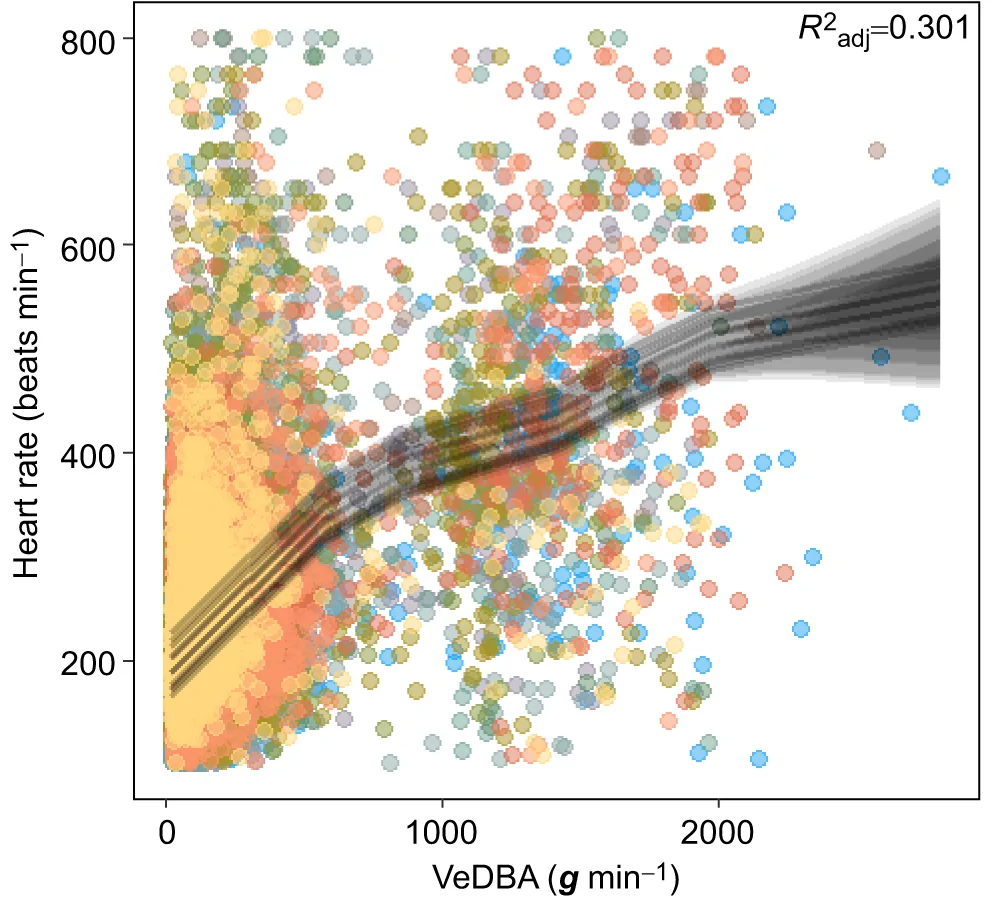
**Model 1.** The relationship between VeDBA (***g*** min^−1^) and heart rate (beats min^−1^). The fitted model is shown in black, with raw data plotted beneath and colored by tag ID. Shaded areas represent ±95% confidence intervals. The adjusted *R*^2^ is displayed in the upper right corner of the panel.

To examine whether the value of VeDBA as a predictor of energy expenditure differs between activity levels (not flying versus flying), we incorporated behavior as an interaction term (model 2, Fig. 5). The inclusion of behavior-specific smooths improved the ability of VeDBA to explain changes in heart rate, with a slight increase in the adjusted *R*^2^ (model 1: *R*^2^_adj_=0.301; model 2: *R*^2^_adj_=0.303). The complexity of the relationship between VeDBA and heart rate also decreased, suggesting a more parsimonious fit (model 1: edf=8.459; model 2: edf=4.596 not flying, 4.885 flying). Incorporating behavior-specific effects for VeDBA improved model fit compared with a model with a single smooth term for all behaviors (χ^2^_5.15_=906,679, *P*<0.001). This suggests that the relationship between VeDBA and heart rate varies across behaviors, and including behavioral states increases the model's accuracy and explanatory power (model 1: AIC=508,411; model 2: AIC=508,289).

**Fig. 5.**
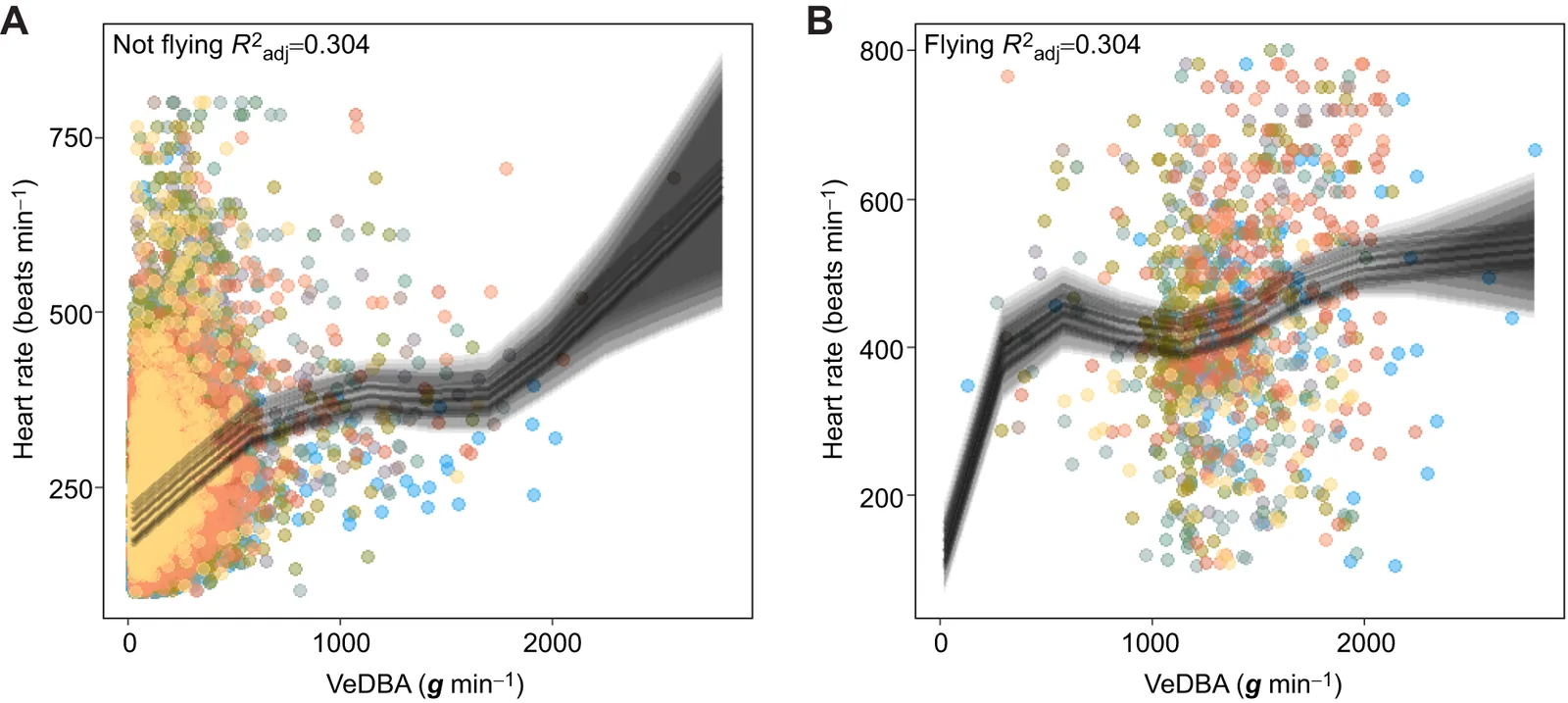
**Model 2.** Heart rate (beats min^−1^) while (A) not flying and (B) flying relative to VeDBA (***g*** min^−1^). The fitted model is shown in black, with raw data plotted beneath and colored by tag ID. Shaded areas represent ±95% confidence intervals. The adjusted *R*^2^ is displayed in the upper left corner of each panel.

To evaluate how VeDBA related to oxygen consumption rate (*V̇*_O_2__), we examined its relationship with energy expenditure (kJ min^−1^), estimated from heart rate (model 3, Fig. S4). A positive relationship between VeDBA and energy expenditure was observed, consistent with greater aerobic demands at higher levels of body acceleration. Although this model explained slightly less variance than the heart rate model (model 1: *R*^2^_adj_=0.301; model 3: *R*^2^_adj_=0.286), and LRT still showed that including the smooth term for VeDBA improved model fit (χ^2^_9.20_=60.52, *P*<0.001). However, the effective degrees of freedom decreased (model 1: edf=8.459; model 3: edf=4.529), indicating a less complex association. When behavior was incorporated into the prediction of energy expenditure (model 4, Fig. S5), similar improvements in model performance were observed as in the heart rate analysis. Specifically, model 4 showed a lower AIC and a higher adjusted *R*^2^ compared with the model without behavioral classification (model 3: AIC=−123,147, *R*^2^_adj_=0.286; model 4: AIC=−123,071, *R*^2^_adj_=0.290). As with the previous models, an LRT confirmed that including behavior as an interaction term improved model fit (χ^2^_5.17_=1.21, *P*<0.001). Additionally, classifying behavior marginally increased the complexity of the smooth terms, as reflected by an increase in edf (model 3: edf=4.529; model 4: edf=4.690 not flying, 4.895 flying).

Behaviorally classified VeDBA combined with behavioral duration and sequence number improved predictions of heart rate (model 5, Fig. 6). This model explained more variance than all previous models (*R*^2^_adj_=0.441) and had a lower AIC compared with the behavior-only model with the additional smooth terms (model 1: AIC=508,411; model 5: AIC=498,446.5), indicating improved model fit and predictive performance. Notably, the complexity of VeDBA as a smooth term in model 5 was reduced relative to model 2 (model 2: edf=4.596 not flying, 4.885 flying; model 5: edf=4.398 not flying, 4.810 flying), demonstrating that including behavioral duration and sequence number refined the relationship between VeDBA and heart rate without sacrificing explanatory power. Both behavioral and sequence number contributed non-linearly to the model (edf=8.960 for duration; edf=8.909 for sequence number), indicating that the influence of recent activity on energy expenditure varies depending on the length of the behavior and temporal context within the foraging bout or commute. Similar improvements in model performance were also observed when using energy expenditure (kJ min^−1^) as the response (model 6: *R*^2^_adj_=0.368; AIC=−128,589). To assess whether the relationship between VeDBA and heart rate differed among individuals, separate models were fit for each bat rather than a single pooled model with an individual-level random effect. The positive relationship between VeDBA and heart rate was consistent across all individuals (Phyl 38: *R*^2^_adj_=0.71; Phyl 34: *R*^2^_adj_=0.70; Phyl 39: *R*^2^_adj_=0.69; Phyl 37: *R*^2^_adj_=0.60; Phyl 29: *R*^2^_adj_=0.57; Phyl 31: *R*^2^_adj_=0.54; Phyl 35: *R*^2^_adj_=0.51; Phyl 32: *R*^2^_adj_=0.50; Phyl 40: *R*^2^_adj_=0.35; Phyl 33: *R*^2^_adj_=0.32; Phyl 36: *R*^2^_adj_=0.28), although model fit varied among bats. To investigate this variation, we examined several potential explanatory factors, including flight sample size, the number and duration of flight bouts, deployment date, and body mass. None of these factors fully accounted for the observed variation in model fit. However, the proportion of high-quality heart rate samples (QI 0–1) recorded for each individual showed a strong association with model performance. A simple linear regression indicated that this variable alone explained 70.2% of the variance in per-individual model fit (*r*=0.838, *P*=0.001) (Fig. S6). Removing the three individuals with the lowest proportions of high-quality heart rate data (Phyl 33, Phyl 36, Phyl 40) from our best performing model (model 5, Fig. 6) improved model performance (*R*^2^_adj_=0.50).

**Fig. 6.**
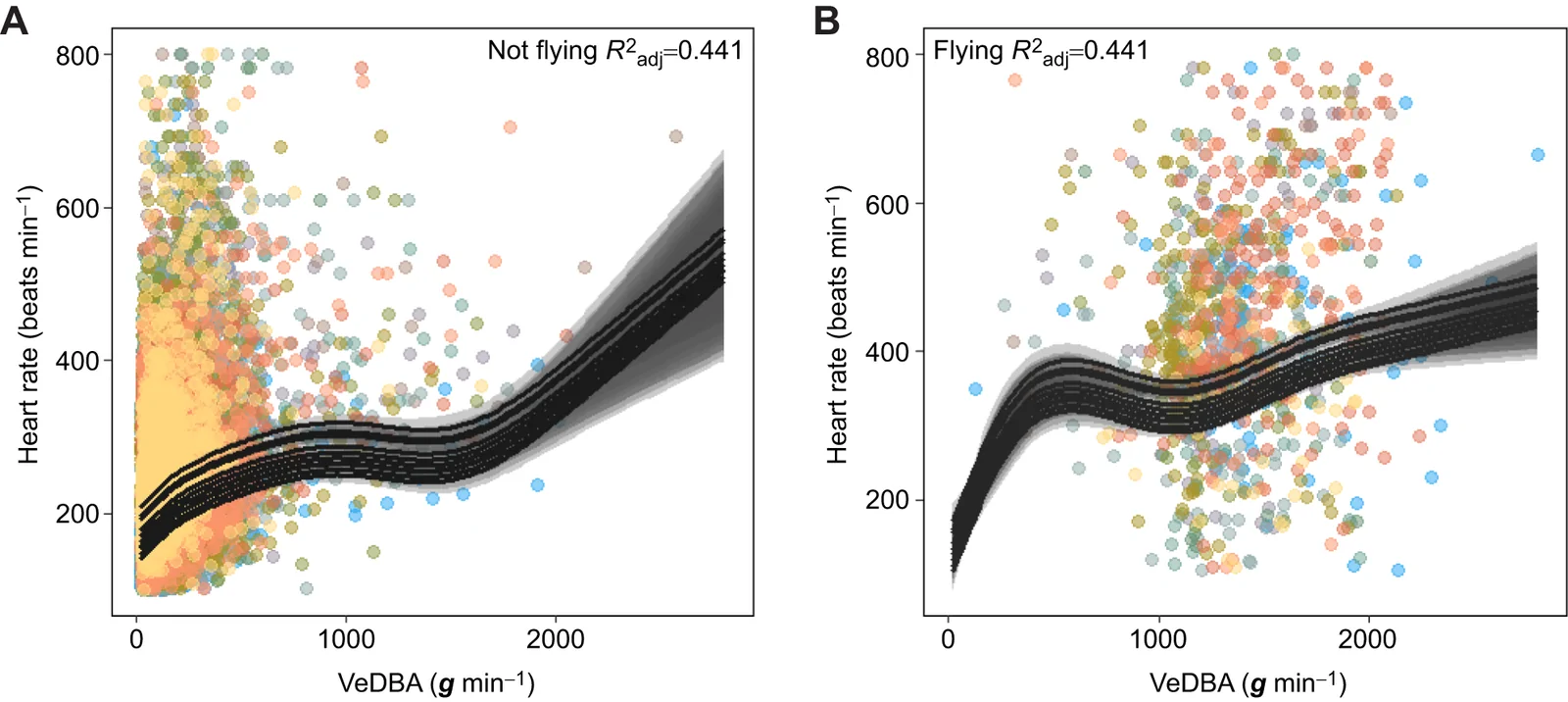
**Model 5.** Heart rate (beats min^−1^) relative to VeDBA (***g*** min^−1^) when behaviorally classified and informed by behavior duration and sequence number, shown for (A) not flying and (B) flying. The fitted model is shown in black, with raw data plotted beneath and colored by tag ID. Shaded areas represent ±95% confidence intervals. The adjusted *R*^2^ is displayed at the top of each panel.

### Airspeed and energy expenditure relationship

To assess how energy expenditure changes with airspeed (m s^−1^), we constructed models (GAMMs) with airspeed as the independent variable (models 7 and 8, Fig. 7; model 8, Fig. S7). Across all models, we observed a negative relationship between airspeed and our metrics of energy expenditure. Both heart rate and its derived energy expenditure estimate (kJ min^−1^) exhibited linear relationships with airspeed (Fig. 7A: edf=1.000; Fig. S7: edf=1.000), whereas the relationship between airspeed and VeDBA was more complex (Fig. 7B: edf=3.011). Airspeed explained the most variability in VeDBA (*R*^2^_adj_=0.314) compared with heart rate (*R*^2^_adj_=0.176) and kJ min^−1^ (*R*^2^_adj_=0.150), indicating that airspeed is most predictive of body acceleration (VeDBA), followed by heart rate and then heart rate-derived energy expenditure (kJ min^−1^).

**Fig. 7.**
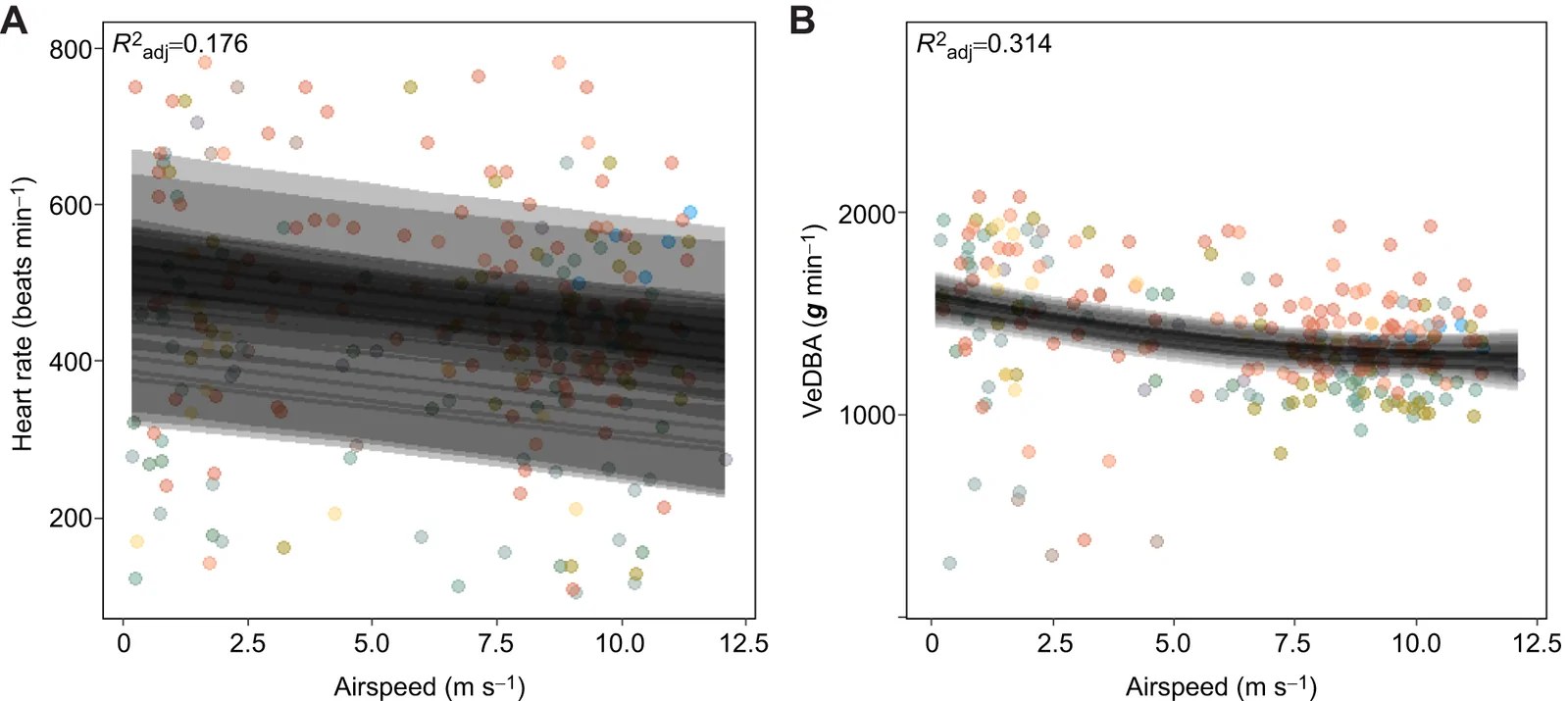
**Models 7 and 8.** Effect of airspeed (m s^−1^) on metrics of energy expenditure. The fitted model is shown in black for (A) Model 7: heart rate (beats min^−1^) and (B) model 8: VeDBA (***g*** min^−1^), with raw data plotted beneath and colored by tag ID. Shaded areas represent ±95% confidence intervals. The adjusted *R*^2^ is displayed in the upper right corner of each panel.

Because flying just below their maximum range speed is predicted to be the most energetically efficient, we expected that these bats would increase their flight speed shortly after take-off. On average, after ∼220 s of flight, bats increased their airspeed above the minimum power speed (6.81 m s^−1^) while staying below their maximum range speed (11.0 m s^−1^), maintaining it within this energetically optimal range for the duration of the flight until landing (Fig. 8). During long-distance flights (those lasting more than 3 min), bats reached an average airspeed of 8.18±2.61 m s^−1^, nearly triple the speed observed during shorter flights, which averaged just 2.07±1.75 m s^−1^.

**Fig. 8.**
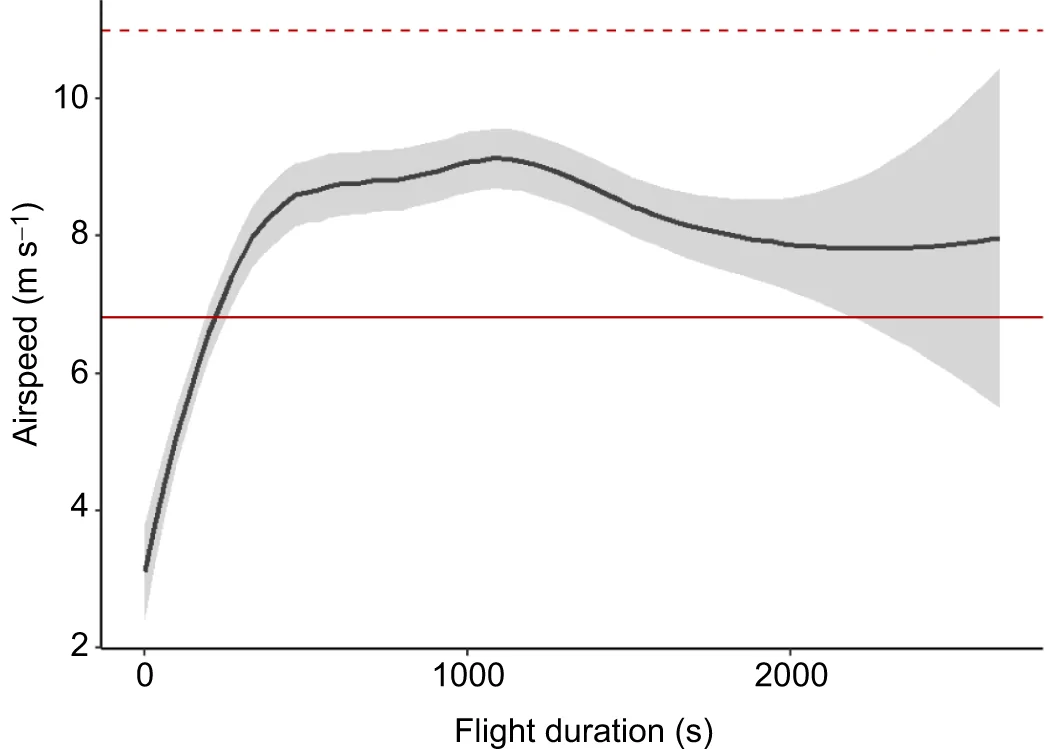
**Average airspeed of *P. hastatus* over the duration of flight.** Minimum power speed is represented by the horizontal solid red line. Maximum range speed is represented by the horizontal dashed line.

## DISCUSSION

Our study provides new insights into the energetics of long-distance flight in bats using heart rate loggers, tri-axial accelerometers and GPS to measure three metrics of energy expenditure – heart rate, VeDBA and airspeed – in free-flying greater spear-nosed bats. VeDBA values represented cumulative activity summed over the 60 s immediately preceding each measurement of heart rate. This allowed us to capture recent locomotor effort rather than instantaneous acceleration at the exact second of heart rate recording. This approach was chosen to better reflect the short-term physiological response of heart rate to recent activity, rather than momentary fluctuations in movement. Through modeling, we revealed positive relationships between heart rate and VeDBA that were improved through behavioral classifications of flight and non-flight, suggesting that the relationship between VeDBA and energy expenditure varies by behavioral state. This improved model fit indicates that fitting a single relationship across multiple behavioral states can obscure underlying energetic relationships. Thus, classifying accelerometry into distinct behavioral states represents an effective first step towards more accurately estimating how bats expend energy across the landscape.

When assessing the performance of accelerometry as a proxy for energy expenditure, it is necessary to consider the medium through which animals move and the context of their locomotion. Bats, for example, rely almost exclusively on flapping flight as their dominant mode of locomotion, which constrains the variability in acceleration patterns and energy expenditure. In contrast, species that travel through multiple media, such as diving or terrestrial birds, often exhibit stronger relationships between dynamic body acceleration and energy expenditure, likely because the resistance encountered in air, in water and on land amplifies mechanical effort and enhances the signal captured by accelerometers (Green et al., 2009; Hicks et al., 2017, 2020; Stothart et al., 2016). Similarly, studies conducted in captivity or on treadmills constrain the animal to simple, repetitive locomotor tasks in a controlled environment, which can inflate the observed relationship between dynamic body acceleration and energy expenditure (Green et al., 2009; Halsey et al., 2008; Miwa et al., 2015; Leimgruber et al., 2023; Williams et al., 2014). Free-ranging animals, by contrast, experience highly variable environmental and behavioral contexts, introducing ‘noise’ that can reduce the apparent correlation between acceleration and energetic output.

Another important factor is the temporal scale at which energy expenditure is assessed. Many previous studies validate accelerometry against doubly labeled water or other methods that integrate energy expenditure over longer periods of time (e.g. 5 min to 1 day+), sacrificing the ability to capture instantaneous fluctuations in energy use (Sutton et al., 2023; Elliot et al., 2013; Hicks et al., 2020; Stothart et al., 2016). Such aggregation generally increases *R*^2^ values because short-term variability is smoothed. In contrast, our study calculated energy expenditure over short, fine-scale bouts, reflecting near-instantaneous energy demands. As a result, we expected lower *R*^2^ values comparatively. However, when energy expenditure was aggregated over a daily scale, our model's predictive power improved substantially (*R*^2^=0.817; Fig. S8), showing that temporal resolution strongly influences observed relationships.

Our best behaviorally informed model still achieved *R*^2^=0.441, which is comparable to previous studies focused specifically on flight: Hicks et al. (2017) reported *R*^2^=0.213 for flight in European shags, and Mizrahy-Rewald et al. (2022) reported *R*^2^=0.61 for northern bald ibis. Other studies either focused on predominantly gliding or soaring flight (Conners et al., 2024; Duriez et al., 2014; Weimerskirch et al., 2016), did not synchronize heart rate and acceleration data (Brown et al., 2022), or averaged energy expenditure over longer periods (Bishop et al., 2015; Brown et al., 2022; Conners et al., 2024; Flack et al., 2020). By capturing short-term, behaviorally classified acceleration and heart rate in free-flying bats, we provide initial relationships between dynamic body acceleration as a proxy for near instantaneous energy expenditure. At the same time, we acknowledge that understanding both within- and among-individual variation in energy distribution is useful for fully resolving the relationship between movement and energy expenditure. Our relatively small sample size limits our ability to properly characterize these sources of variation or assess how individual differences in morphology, physiology or behavior may influence the observed relationships. Future studies with larger sample sizes and repeated measures across individuals would help clarify these effects and determine how consistent these relationships are across individuals versus being driven by individual-specific strategies.

In species exhibiting complex locomotion, incorporating additional behavioral and temporal context into models can improve our ability to link movement-based metrics with associated energy costs. Consistent with this, VeDBA remained the strongest predictor of energy expenditure when combined with additional information on flight duration and sequence number (as evidenced by lower AIC and higher *R*^2^_adj_), indicating that heart rate is influenced not only by mechanical effort, but also by the temporal context of activity. The inclusion of these terms suggests that energy expenditure changes over the course of a flight and varies across successive bouts, better capturing aspects of dynamic flight strategies, such as changes in airspeed, and physiological state such as fatigue, recovery or sustained effort that are not fully explained by VeDBA alone. This strengthens the biological interpretation by demonstrating that heart rate reflects both immediate and locomotor activity and longer-term temporal dynamics, and that incorporating these temporal components provides additional explanatory power to acceleration-based metrics.

Additionally, when assessing how both of our metrics of energy expenditure responded to a third metric of energy expenditure, VeDBA showed a stronger and more direct relationship with airspeed than did heart rate, which is expected given the mechanistic basis of these metrics. VeDBA reflects instantaneous mechanical activity derived from body acceleration and therefore responds immediately to changes in flight dynamics such as airspeed. In contrast, heart rate represents a physiological response that integrates energetic demand over time and is subject to inherent delays owing to cardiovascular and metabolic processes. These temporal lags mean that heart rate may not fully capture rapid fluctuations in flight effort at fine temporal scales, particularly during dynamic changes in airspeed. As a result, VeDBA more closely tracks the mechanical power requirements of flights, whereas heart rate provides a temporally smoothed measure of overall energy expenditure. This distinction likely contributes to the tighter relationship observed between VeDBA and airspeed. Additionally, this relationship would likely become even stronger with finer-scale behavioral classification. For example, elevated heart rates often persist briefly after landing, reflecting recovery processes rather than active locomotion, which can decouple heart rate from both airspeed and instantaneous mechanical effort. Incorporating more detailed behavioral states (e.g. taking off, active flight, landing, post-flight recovery) would help isolate periods of true locomotor activity and reduce this temporal mismatch, and potentially strengthen the observed relationship between these variables.

In addition to these temporal and mechanistic differences between VeDBA and heart rate, we also found some variation among individuals in how well the model described the relationship between VeDBA and heart rate. This variation was closely related to the proportion of high-quality heart rate measurements available for each bat. Individuals with fewer high-quality measurements tended to have poorer model fit, which suggests that differences in heart rate data quality were largely responsible for the variation among individuals rather than differences in the underlying relationship between VeDBA and heart rate. Individuals with weaker relationships would have likely shown stronger associations with longer tracking durations and a greater number of high-quality heart rate measurements. This is supported by the improvement in overall model fit when the three individuals with the poorest-fitting models were excluded. This improvement provides further support for the use of VeDBA as a proxy for energy expenditure, while also highlighting the importance of obtaining sufficient high-quality heart rate data when using it to validate activity-based measures of energy expenditure. Next, we examined how the relationship between VeDBA and heart rate varied with airspeed, and whether it followed the expected pattern of energy expenditure across flight speeds.

Airspeed itself showed a non-linear influence on the model (edf=3.011), indicating that its effect on energy expenditure is better described by a smooth curve rather than a simple linear relationship. In fact, if dynamic body acceleration serves as a true reflection of energy expenditure (Usherwood et al., 2011; Spivey and Bishop, 2013), aerodynamic theory predicts that some curvilinear relationship between VeDBA and airspeed should be observed within our flight data (O'Mara and Dechmann, 2023; Rayner, 1999; Pennycuick, 2008). This curvilinear association was evident in the relationship between VeDBA and airspeed, which closely followed the mechanical power curves predicted for this species (O'Mara and Dechmann, 2023) (Fig. S9). The power curve for *P. hastatus* demonstrates how energy expenditure is not linear across flight speeds, but instead increases at both low and high airspeeds. At lower speeds, such as taking off or hovering flight, bats must generate substantial lift to remain airborne, requiring greater muscular effort. Beyond this, our models show that energy expenditure continually decreases as airspeed increases until reaching the estimated minimum power speed for *P. hastatus* (Fig. 7). This is similar to the general pattern of decreasing mechanical power with increasing airspeed found across flying animals (Fig. S9) (O'Mara and Dechmann, 2023; Pennycuick, 2008).

At increasingly higher flight speeds beyond the maximum range speed, rising parasitic drag substantially increases energy expenditure. As a result, intermediate speeds offer a compromise between low energetic cost and effective distance coverage. This balance between energetic efficiency and travel demands likely informs bats' use of different airspeeds in response to behavioral goals. Bats have even been shown to modify their airspeed depending on the behavioral context and surrounding environmental conditions (Grodzinski et al., 2009; Sapir et al., 2014). The deliberate use of distinct airspeeds by *P. hastatus* is suggested by the bimodal distribution of observed flight speeds, likely reflecting shifts in behavior based on ecological or functional needs. In this case, the two peaks may reflect functionally different flight strategies, such as commuting versus foraging, supporting the idea that *P. hastatus* selects airspeeds based on task-specific energetic demands. Consistent with this, our findings show that *P. hastatus* rapidly adjusted their airspeed above their minimum power speed early in their flight during long-distance commutes and sustained it within an energetically efficient range for the duration of the trip. This rapid adjustment suggests that bats may anticipate the energetic demands of flight in advance and modify their airspeed depending on the distance to be covered or the purpose of travel. This ability to anticipate and modulate flight behavior may be especially important given the steep energetic costs associated with slower flight speeds. The sharp increases observed in VeDBA as airspeed drops below 6 m s^−1^ demonstrate that even subtle changes in flight speed can carry disproportionate energetic consequences, reinforcing the importance of integrating airspeed into models of energy expenditure. Ultimately, contextualizing VeDBA with behavior and airspeed enables us to explore how and why individual bats distribute their energy expenditure across different flight strategies.

As environmental pressures intensify and habitats become increasingly fragmented, understanding how animals navigate energetic trade-offs in their movement decisions will be critical for predicting species' resilience and informing conservation strategies (Ellis-Soto et al., 2025). Because energetic costs likely influence decisions about when, where, why and how to move, such metrics should prove to be valuable when trying to understand the drivers of movement strategies. Together, our results suggest that VeDBA has a close predictive relationship with energy expenditure in bats, and that this less-invasive measure of energy expenditure and behavioral insight can be used to understand broad-scale energy expenditure and its ecological context for wild bats. These findings highlight the potential of accelerometry-based metrics to inform conservation by linking movement, physiology and habitat use. In doing so, they open up new possibilities for understanding the energetic constraints shaping bat ecology and how bats adjust their strategies to shifting resource landscapes, which will be valuable for predicting how these animals will cope with a rapidly changing world (Kohles et al., 2024; O'Mara et al., 2019).

## Supplementary Material

10.1242/jexbio.251521_sup1 Supplementary information
