# Supplementary material for: Heart rate supports accelerometry as a reliable predictor of energy expenditure in bats
Source: J Exp Biol. 2026 Oct 22;229(20):jeb251521. doi: 10.1242/jeb.251521 (PMC13723403; doi:10.1242/jeb.251521)
Supplement: Supplementary file 1 [file jexbio-229-251521-s1.pdf]

Table S1. Characteristics of greater spear-nosed bats tracked in this study. Information included is the weight, sex, and relative age of the bat - noted as adult (A) or subadult (SA), forearm length, reproductive status—with all being non-reproductive (NR), GPS identification number (GPS ID), heart rate logger identification number (HRL ID), and number of nights they were tracked.

Mass (g)	Sex	Age	Forearm (mm)	RS	GPS ID	HRL ID	Nights
112	F	A	91.00	NR	Phyl32	1864UHL	5
118	F	A	93.20	NR	Phyl34	1863UHL	5
110	F	A	89.43	NR	Phyl 30	1859UHL	5
113	F	A	94.64	NR	Phyl 37	1855UHL	5
110	F	A	91.07	NR	Phyl 31	1857UHL	5
111	F	A	92.05	NR	Phyl 38	1858UHL	4
117	F	A	93.45	NR	Phyl 36	1854UHL	5
128	F	A	90.55	NR	Phyl 35	1865UHL	2
146	M	A	97.24	NR	Phyl 29	1861UHL	5
123	F	SA	93.30	NR	Phyl 33	1856UHL	5
126	F	A	93.20	NR	Phyl 40	1860UHL	5
112	F	A	90.60	NR	Phyl 39	1862UHL	3

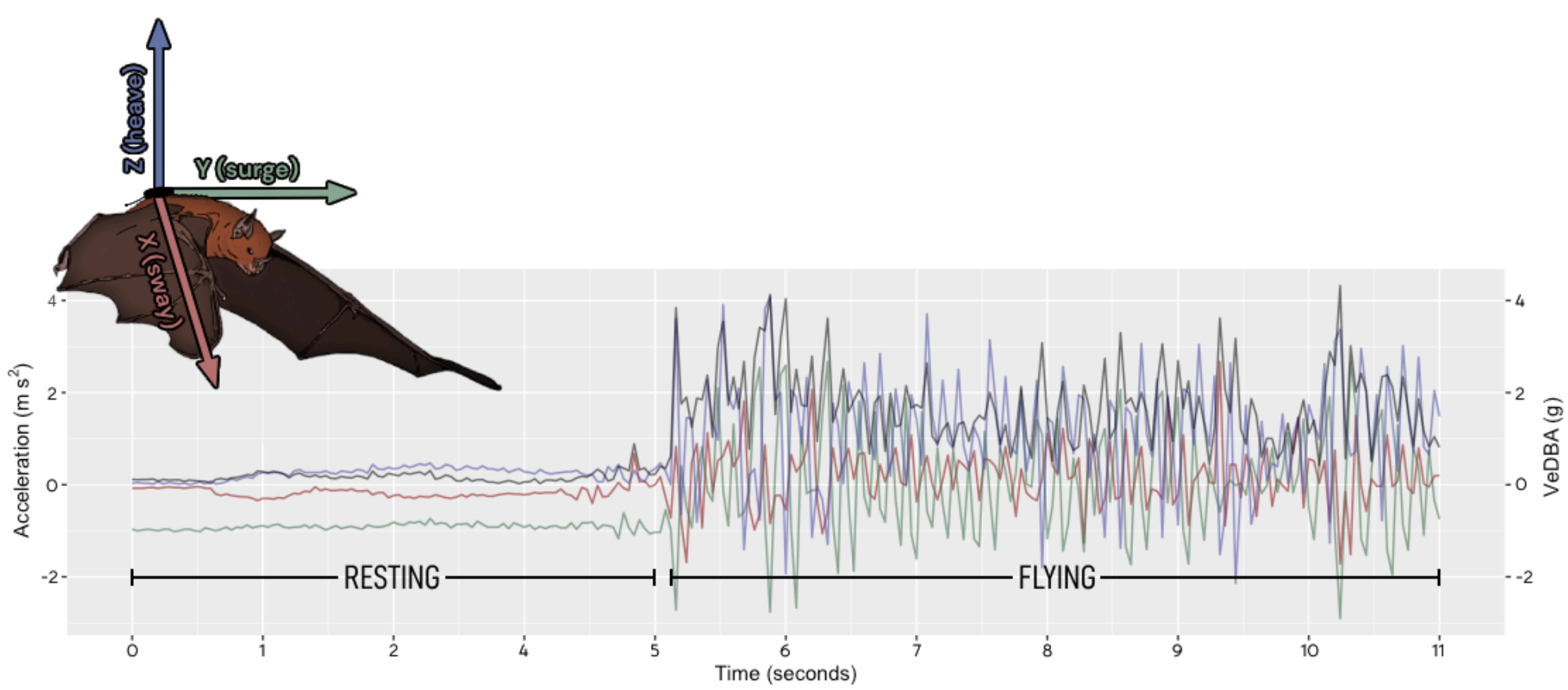

Fig. S1. Tri-axial accelerometry data with corresponding VeDBA estimated from it. The data are derived from 11 seconds of tri-axial accelerometry measurements from the moment a greater spear-nosed bat takes off for flight, showing the significant changes seen in the acceleration of each axis, and the value of VeDBA reflected by it. Tri-axial accelerometers record acceleration in all three axes as shown by the bat in the upper corner of the figure. The X axis (sway) is represented by red, the Y axis (surge) by green, the Z axis (heave) by blue, and VeDBA by black.

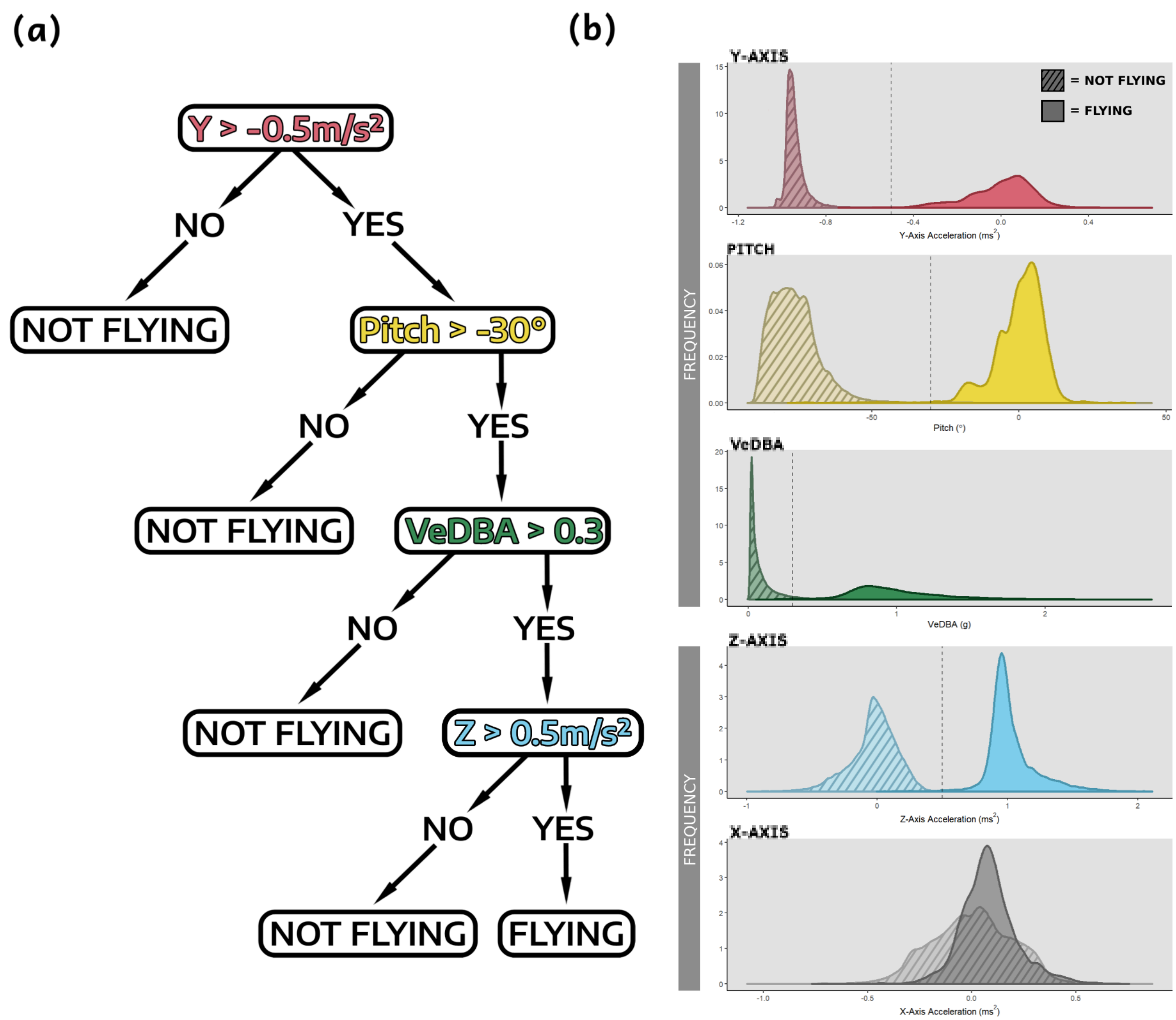

Fig. S2. Accelerometry-derived metrics used to classify two distinct behaviors in greater spear-nosed bats. (a) Behavioral decision tree showing threshold and the hierarchical classification used to identify flight. Decisions are ranked by their relative importance, with the most influential at the top. As the data move down the tree, if all conditions are met, the behavior is classified as flight. **(b)** For each metric, the color used in the decision tree matches the color of its corresponding distribution on the right. Flight distributions are shown in solid colors, while non-flight distributions are represented by diagonal hatching. Vertical dashed lines indicate meaningful thresholds distinguishing the two behaviors. The x-axis distribution at the bottom, shown in grey, demonstrates that not all metrics are useful to the model.

Journal of Experimental Biology • Supplementary information

Table S2. Energy expenditure and morphological data of individual bats. Information includes sex, body weight (M_b), heart weight (M_h), average rate of oxygen consumption during flight ($\bar{X}\ \dot{V}O_{2F}$), average energy expenditure ($\bar{X}\ EE_F$) and VeDBA ($\bar{X}$ $VeDBA_F$) during flight, and heart rate logger ID.

Sex	M_b (kg)	M_h (kg)	$\bar{X}\ \dot{V}O_{2F}$ (ml min^{-1})	$\bar{X}\ EE_F$ (kJ min^{-1})	$\bar{X}$ $VeDBA_F$ (*g* min^{-1})	GPS ID	HRL ID
F	0.112	0.00110	16.22	0.342	1479.13	Phyl32	1864UHL
F	0.118	0.00116	9.93	0.210	1395.72	Phyl34	1863UHL
F	0.113	0.00111	9.80	0.207	1343.01	Phyl 37	1855UHL
F	0.110	0.00108	8.27	0.175	1309.22	Phyl 31	1857UHL
F	0.111	0.00109	12.26	0.259	1572.64	Phyl 38	1858UHL
F	0.117	0.00115	8.58	0.181	1263.79	Phyl 36	1854UHL
F	0.128	0.00125	8.43	0.178	1274.95	Phyl 35	1865UHL
M	0.146	0.00143	12.12	0.256	1640.57	Phyl 29	1861UHL
F	0.123	0.00121	8.40	0.177	1384.44	Phyl 33	1856UHL
F	0.126	0.00123	6.04	0.128	1292.59	Phyl 40	1860UHL
F	0.112	0.00110	10.67	0.225	1422.22	Phyl 39	1862UHL

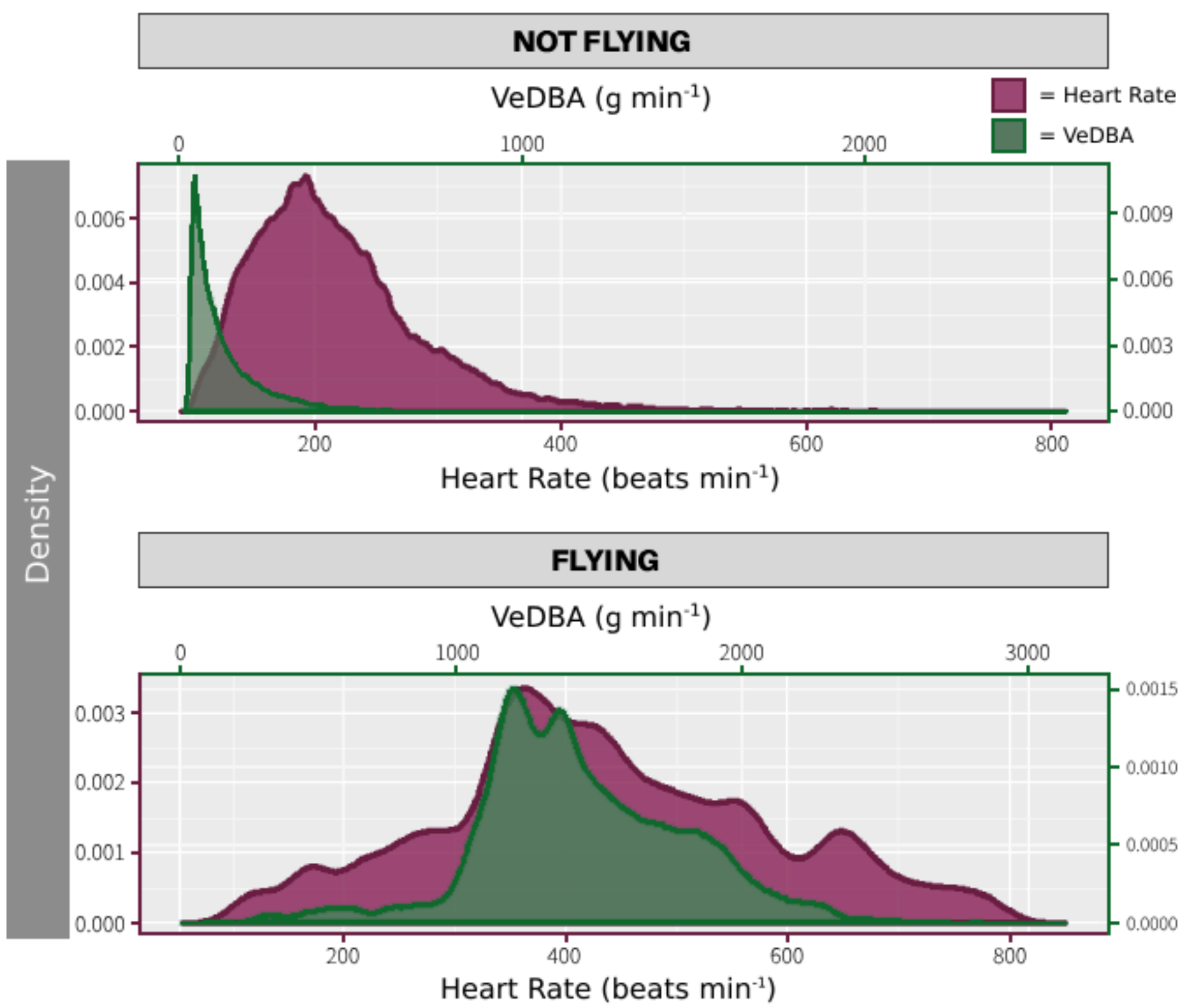

Fig. S3. Density plots illustrating the distribution of heart rate and VeDBA during periods of non-flight and flight across all bats. Heart rate and its respective scales are indicated by the violet coloration. VeDBA and its scales are indicated by the green coloration. While not flying, energy metrics become aggregated at low levels. While during flight, the metrics become widely distributed at higher values.

Journal of Experimental Biology • Supplementary information

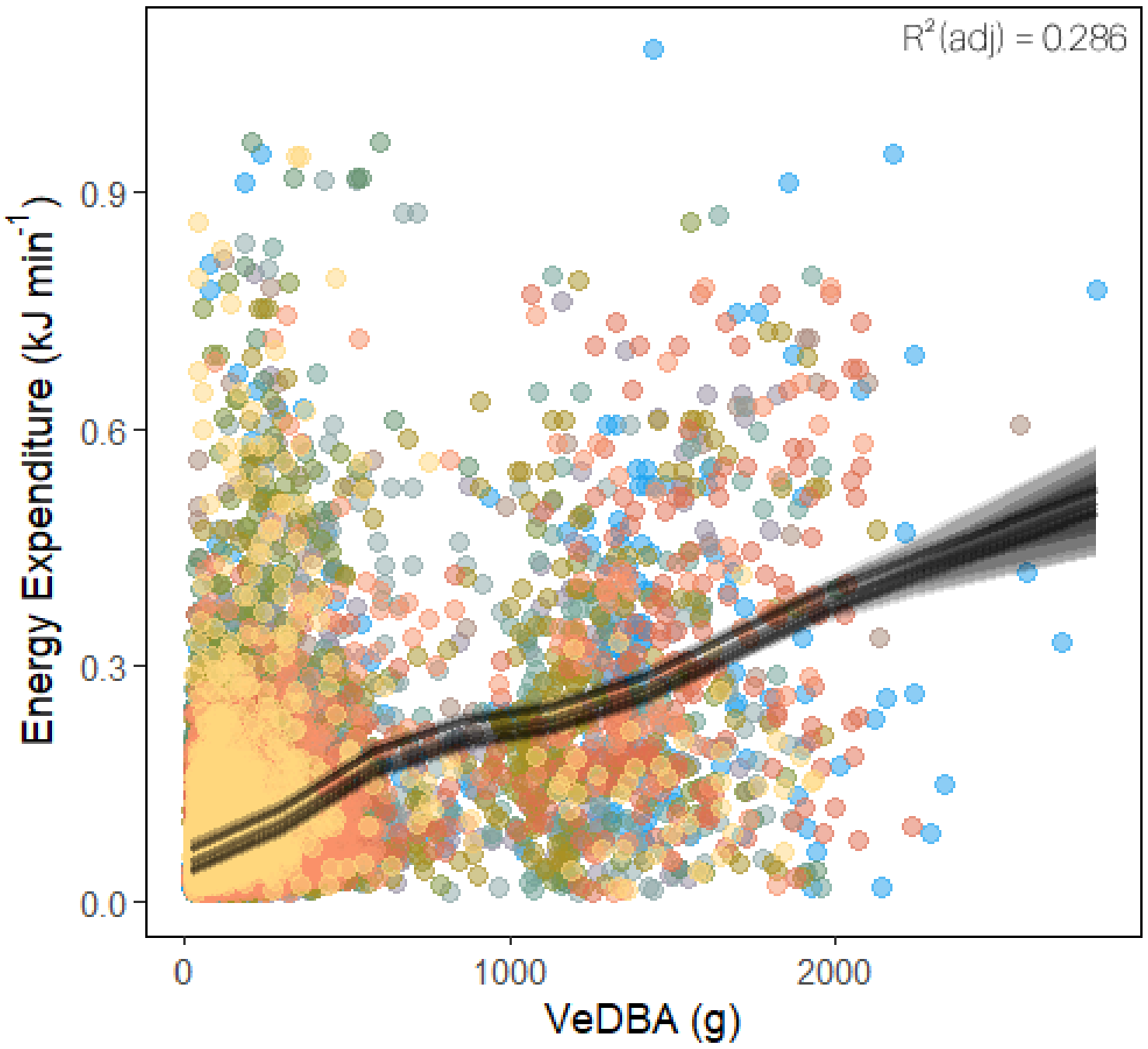

Fig. S4. Model 3. The relationship between VeDBA (g min^{-1}) and energy expenditure (kJ min^{-1}). The fitted model is shown in black, with raw data plotted beneath and colored by tag ID. Shaded areas represent ± 95% confidence intervals. The adjusted R^2 is displayed in the upper right corner of the panel.

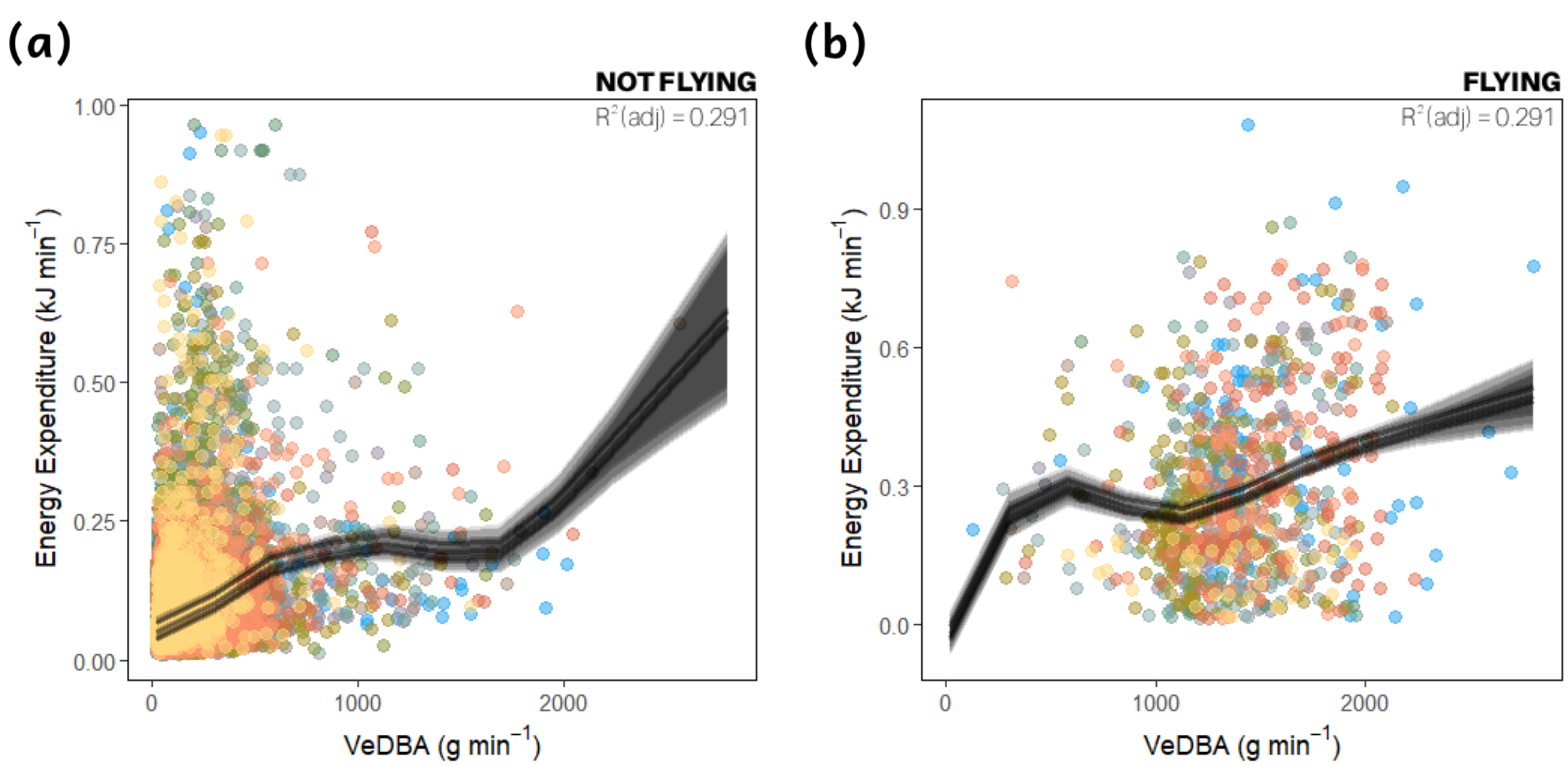

Fig. S5. Model 4. Energy expenditure (kJ min^{-1}) during non-flight (a) and flight (b) relative to VeDBA (*g* min^{-1}). The fitted model is shown in black, with raw data plotted beneath and colored by tag ID. Shaded areas represent ± 95% confidence intervals. The adjusted R^2 is displayed in the upper right corner of each panel.

Journal of Experimental Biology • Supplementary information

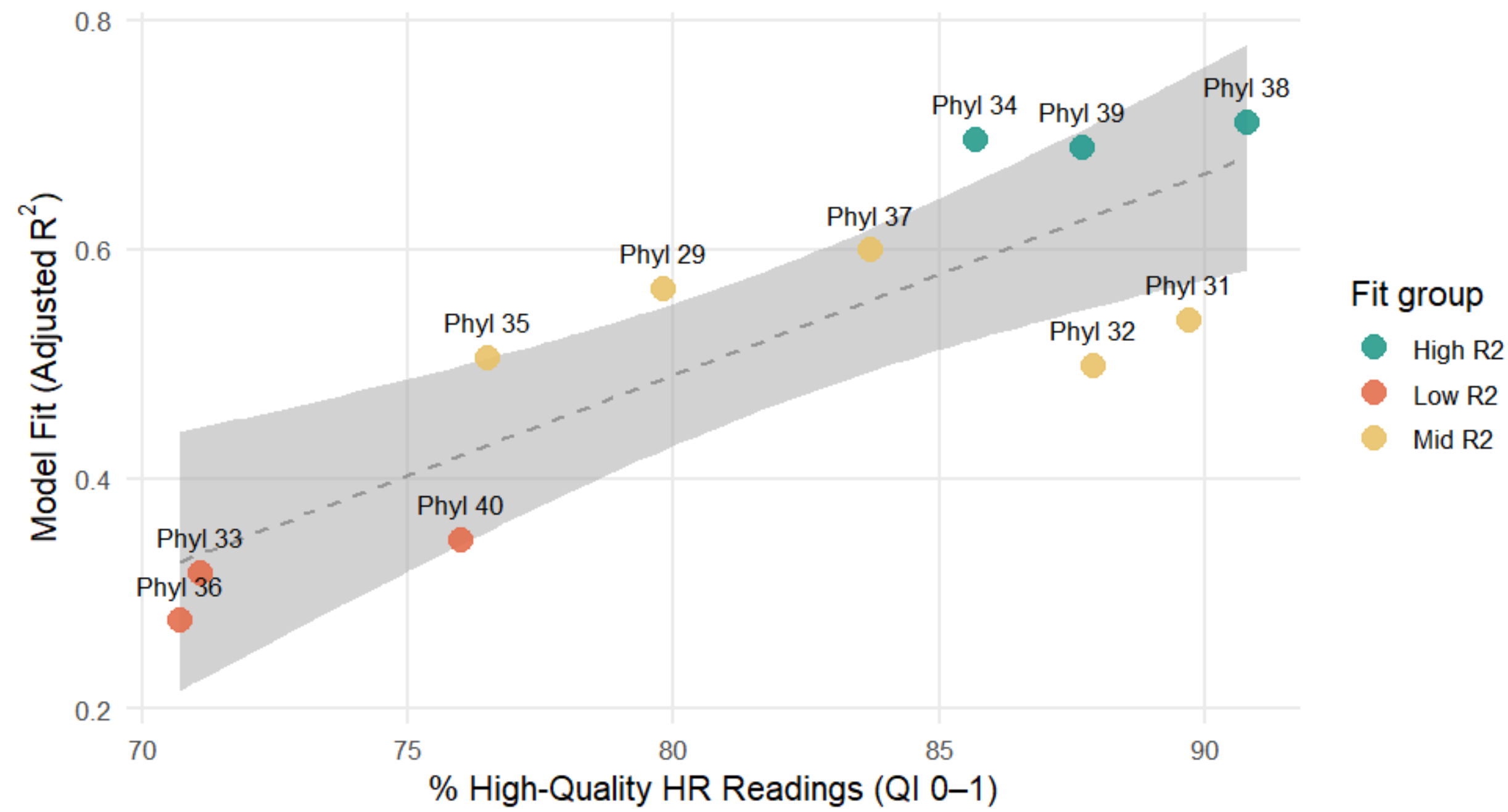

Fig. S6. Relationship between the proportion of high-quality heart rate readings (QI 0-1) and model fit (adjusted R^2) for each bat, with models fit separately per individual. Each point represents one bat, color by relative model fit (low, mid, high R^2), with the dashed line and shaded band showing the linear trend and ± 95% confidence interval. Individuals with a lower proportion of high-quality heart rate data tended to show poorer model fit, suggesting that variation in data quality, rather than fundamental differences in the underlying VeDBA-heart rate relationship, was a primary driver of among-individual variation in model performance.

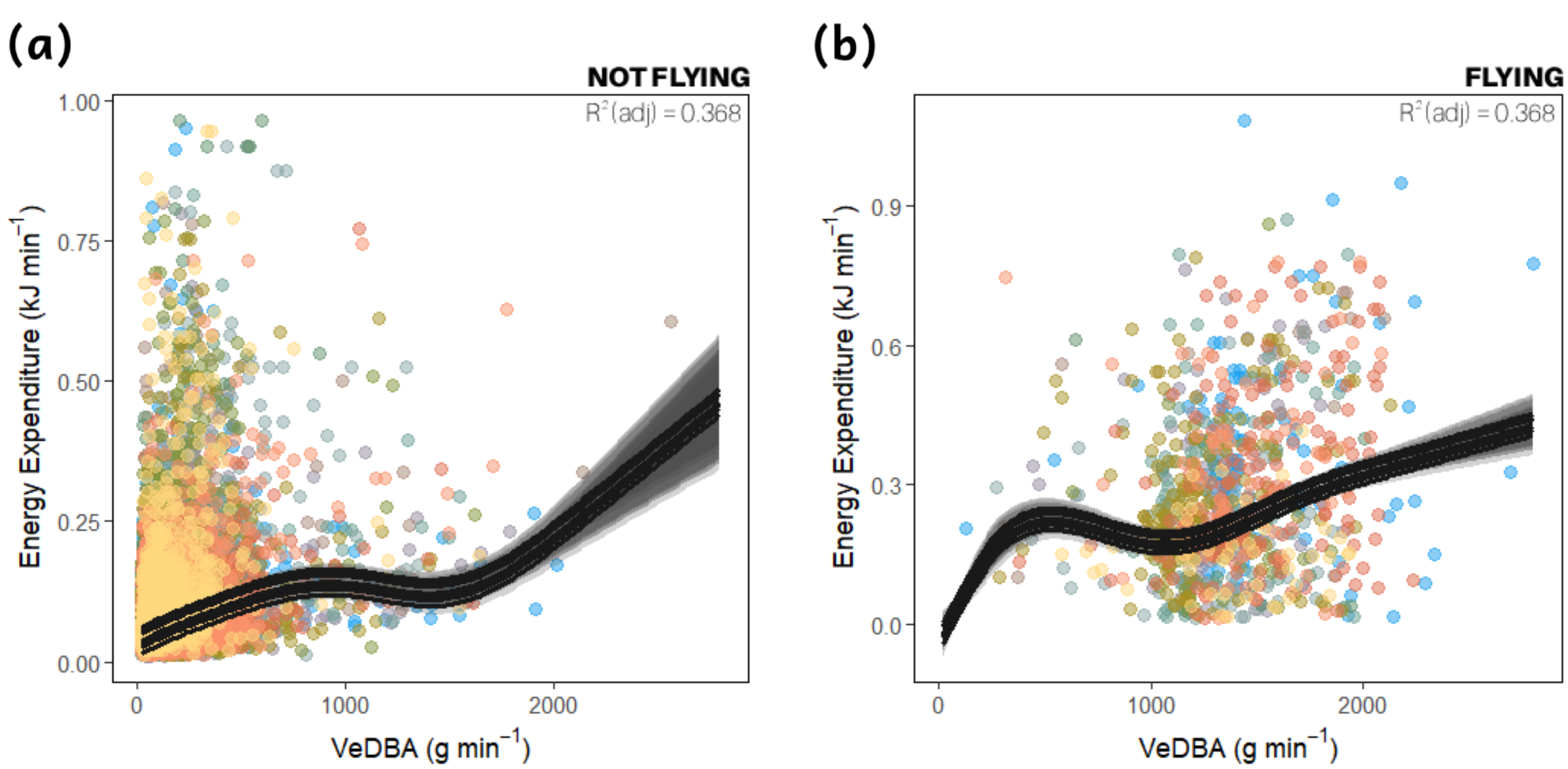

Fig. S7. Models 6. Energy expenditure (kJ min^{-1}) relative to VeDBA (g min^{-1}) when behaviorally classified and informed by behavior duration and sequence number, shown for (a) not flying and (b) flying. The fitted model is shown in black, with raw data plotted beneath and colored by tag ID. Shaded areas represent ± 95% confidence intervals. The adjusted R^2 is displayed in the upper right corner of each panel.

Journal of Experimental Biology • Supplementary information

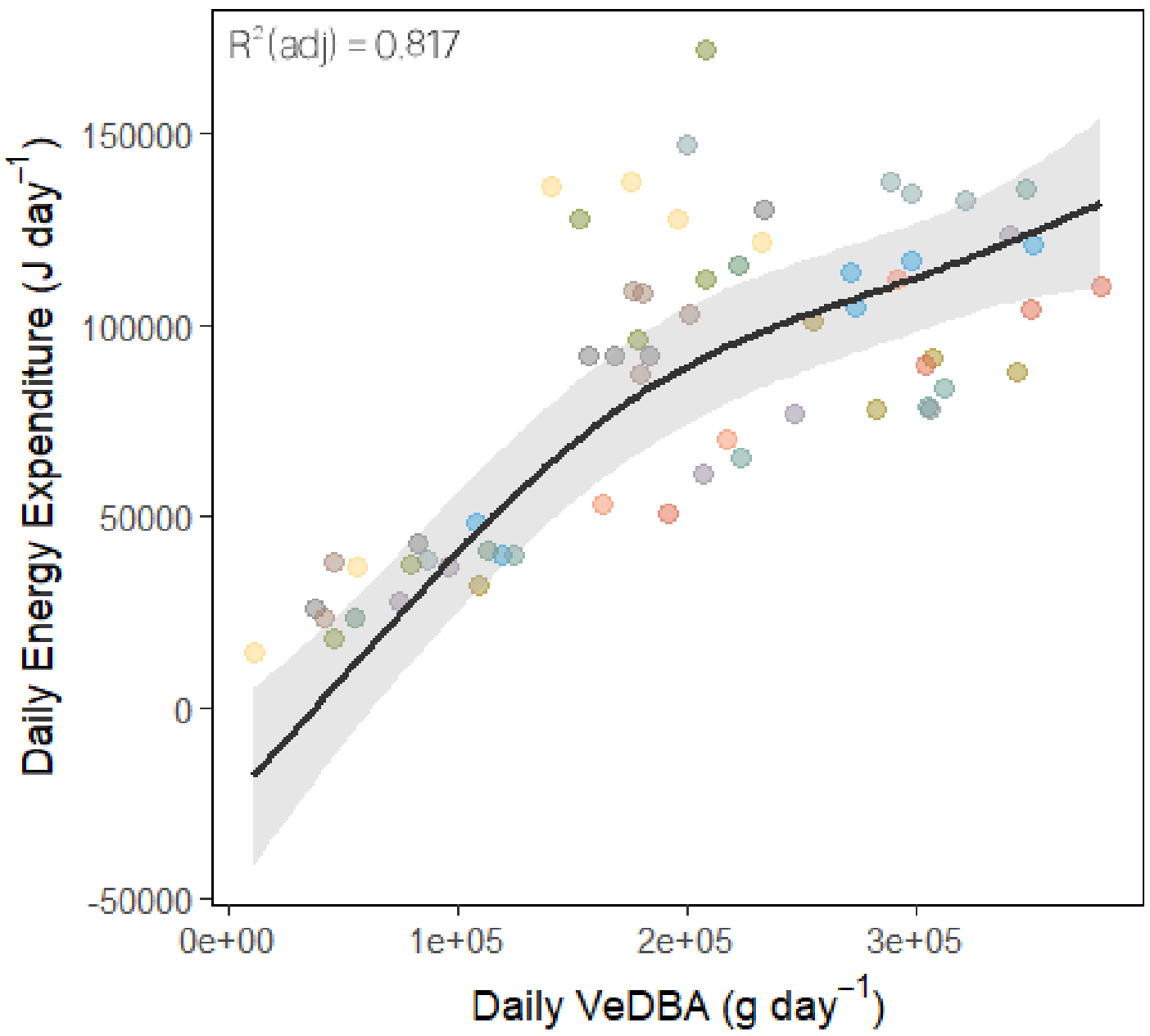

Fig. S8. Daily energy expenditure (J day^{-1}) relative to cumulative daily VeDBA (g day^{-1}). The fitted generalized additive model is shown in black, with raw data plotted beneath and colored by tag ID. Shaded areas represent ± 95% confidence intervals. The adjusted R^2 is displayed in the upper left corner.

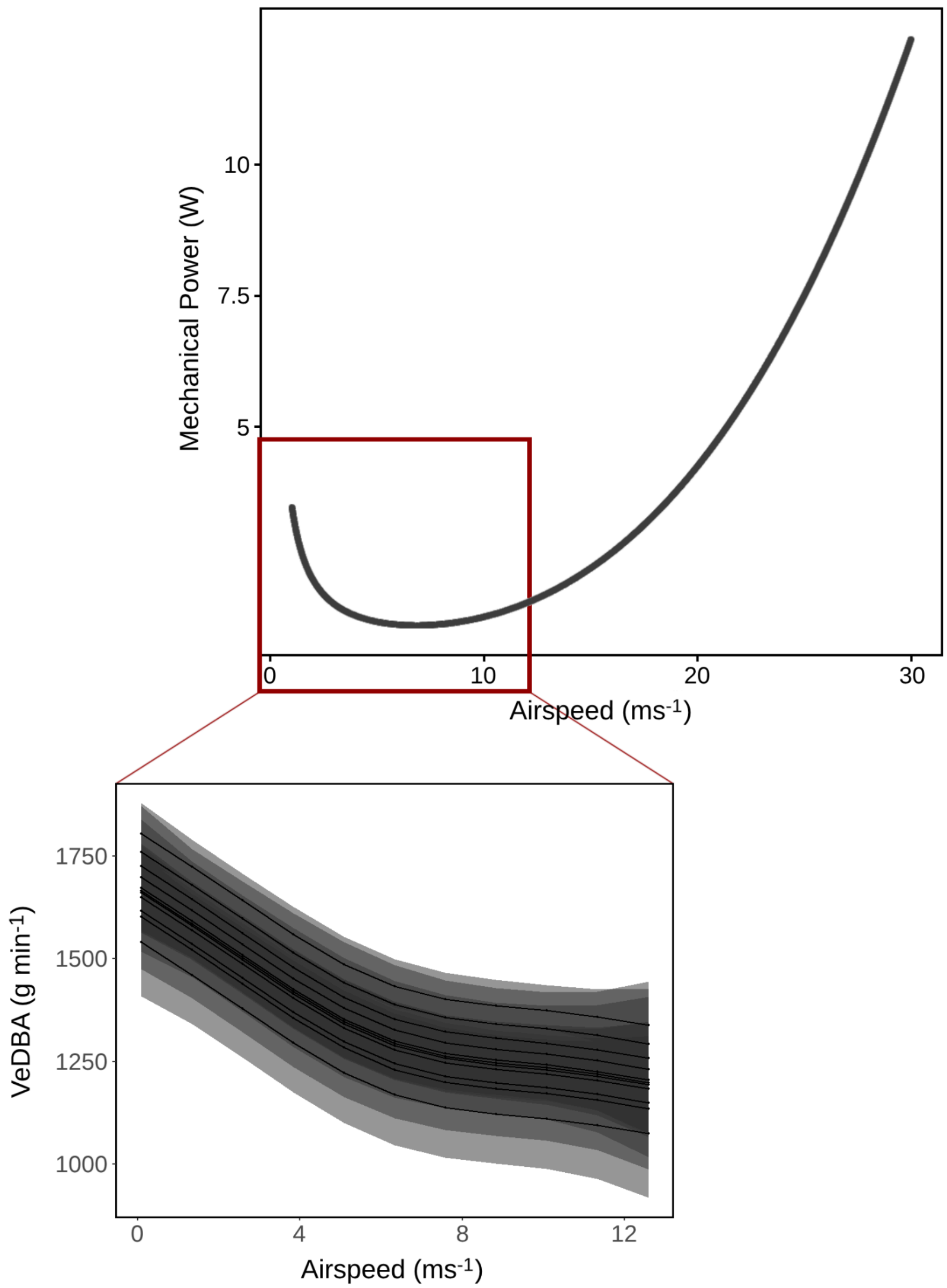

Fig. S9. The estimated power curve of greater spear-nosed bats (top), with an inset (bottom) of the observed energy expenditure of *P. hastatus* across various flight speeds. The red box represents the general position of the observed energy costs relative to the entire power curve estimate.

Table S3. Summary of the symbols, units, and definitions of variables, metrics, and calculations applied in this study. The table provides a reference for all parameters used in the estimation and analysis of energy expenditure in greater spear-nosed bats.

Symbol	Units	Meaning
A_X, A_Y, A_Z	m s^2	Raw acceleration of each axis
S_X, S_Y, S_Z	m s^2	Static acceleration of each axis
θ	°	Pitch angle
D_X, D_Y, D_Z	m s^2	Dynamic acceleration of each axis
VeDBA	g	Vectorial Dynamic Body Acceleration
$\bar{X}$ $VeDBA_F$	g min^{-1}	Mean rate of estimated energy expenditure during flight
EE_{hr}	kJ min^{-1}	Heart rate-derived estimate of energy expenditure
$\bar{X}$ EE_F	kJ min^{-1}	Mean rate of energy expenditure during flight
X, Y, Z	m s^2	Axes of acceleration in three perpendicular planes
$\dot{V}O_2$	ml min^{-1}	Rate of oxygen consumption
$\bar{X}$ $\dot{V}O_{2\,F}$	ml min^{-1}	Mean rate of oxygen consumption during flight
M_b	kg	Body mass
M_h	kg	Heart mass
f_H	beats min^{-1}	Heart beat frequency
U_{mp}	m s^{-1}	Minimum power speed
U_{mr}	m s^{-1}	Maximum range speed

Journal of Experimental Biology • Supplementary information
